# Unveiling Unusual Reactivity of SO_2_ and Unusual Type of S–X Long Bonds

**DOI:** 10.1021/acs.inorgchem.5c02435

**Published:** 2025-07-08

**Authors:** Shuai Ma, Longfei Li, Xiaofeng Xie, Peng Wang, He Bai, Kan Yang, Xueqing Song, Henry F. Schaefer

**Affiliations:** † College of Pharmacy, Key Laboratory of Pharmaceutical Quality Control of Hebei Province, Key Laboratory of Medicinal Chemistry and Molecular Diagnosis of Ministry of Education, 56667 Hebei University, Baoding 071002, Hebei, P. R. China; ‡ Center for Computational Quantum Chemistry, 1355 University of Georgia, Athens, Georgia 30602, United States

## Abstract

A new reactivity of SO_2_ to form unusual stable M–X–SO_2_ (X = F, Cl, H) complexes is unveiled in this study. Moreover, a new type of S–X long bonds, which are significantly longer than traditional S–X covalent bonds, has been discovered. The *P,N*-ligated Ni–F complex model **1A** can bind a SO_2_ molecule through the new F–S long bond (2.207 Å), and a stable Ni–F–SO_2_ complex **1B** is generated, being exergonic by 2.2 kcal/mol. According to natural localized molecular orbital analysis, the new S–F long bond has a unique p­(F) → π*­(O=S=O) bonding interaction, which is shown to arise from the long S–F length. In comparison, the strength of the new F–S long bond (−2.2 kcal/mol) is found to be significantly stronger than common noncovalent interactions such as the hydrogen and halogen bond. The substituent modulations suggest that the electron-donating groups can increase the strength of new F–S bonds and enhance binding free energies Δ*G*_bind_. The scope of possible M–X complexes was explored, and various metals and X (F, Cl, and H) ligands were found to form stable M–X–SO_2_ complexes. Specifically, the anionic M–X complexes display much higher Δ*G*_bind_ values, ranging from −8 to −10 kcal/mol. The study paves the way for a green, recyclable, and adjustable SO_2_ absorption method.

## Graphical abstract

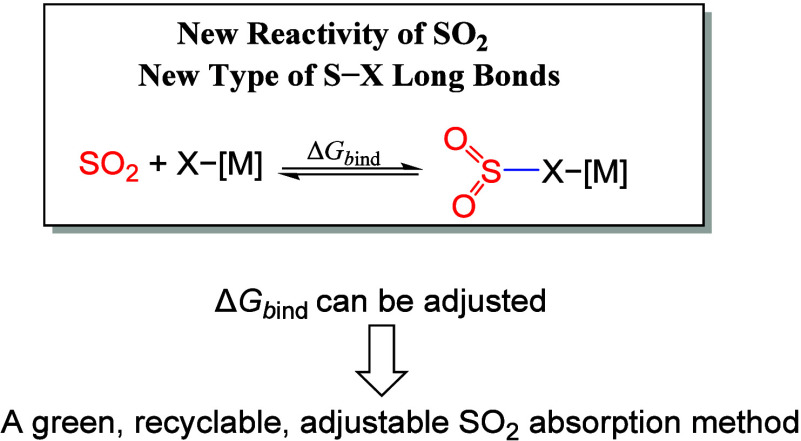

## 1 Introduction

From the combustion of fossil fuels, SO_2_ is the primary atmospheric pollutant contributing to acid rain and haze,^1^ and the mitigation of SO_2_ emissions has long provided a critical focus in chemistry.^2^ The conventional SO_2_ capture approaches are based on limestone,^3^ porous materials,^4,5^ organic solvents,^6,7^ and ionic liquids (Scheme 1).^8–11^ These conventional methods are limited by harmful byproducts,^12^ low selectivity,^13^ high costs,^14,15^ high energy consumption,^16^ unavoidable absorbent volatilization,^17^ or complex synthesis.^18–20^ Furthermore, an efficient SO_2_ adsorbent should be green and recycled in terms of green chemistry.^21^ Organometallic complexes have been applied in nitrogen fixation,^22^ hydrogen storage,^23^ CO reduction,^24^ and CO_2_ reduction^25^ with high efficiency, selectivity, tunability, and environmental friendliness. However, the use of organometallic complexes for SO_2_ fixation has not yet been reported. With the increasingly stringent SO_2_ emission standards,^26^ there is a growing need to develop new chemistry or bonding interactions for achieving efficient organometallic complexes based SO_2_ fixation.^27^

**Scheme 1:**
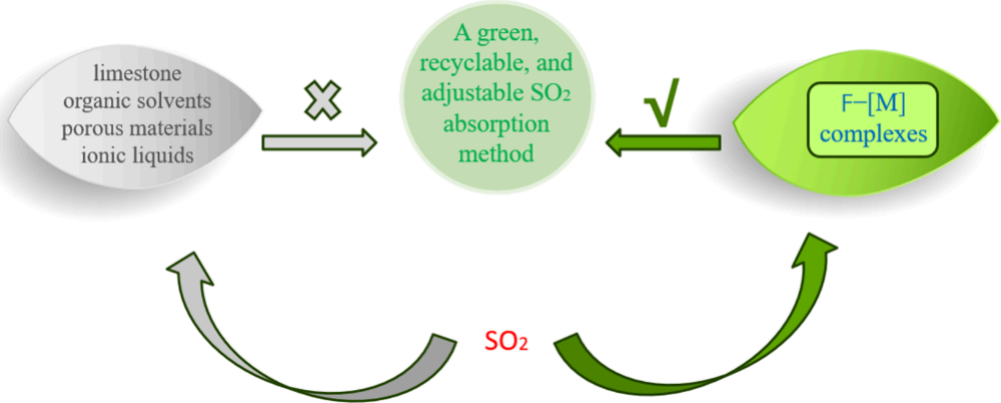
Comparison of SO_2_ Absorption Methods

Weak interactions, such as hydrogen bonds,^28^ halogen bonds,^29–32^ π–π,^33^ CH−π,^34^ cation−π,^35^ anion−π,^36,37^ chalcogen bond,^38^ and hydrophobic interactions,^39^ are fundamental to supramolecules, catalysis chemistry, materials, and biochemistry.^40–42^ The S atoms in sulfur-containing compounds are important hydrogen bond acceptors. For example, the S···H–O and S···H–N hydrogen bonds are crucial for folded structures of protein.^43–45^ Furthermore, S···H–N hydrogen bond interactions have been utilized in the metal catalysts to increase catalytic reactivities.^46,47^ In contrast, there are few reports on weak interactions from the S atom in SO_2_. In 2009, Prasad and Senapati compared the absorptivity of SO_2_, CO_2_, and N_2_ gas molecules in ionic liquids and suggested that the high solubility of SO_2_ in ionic liquids can be explained by dipole–dipole interactions.^48^ In 2021, Kitzmiller and co-workers studied the HOX···SO_2_ (X = F, Cl, Br, I) binary complexes (Scheme 2) and found that SO_2_ can form hydrogen interactions with HOX complexes in which X···S lengths are all more than 3.1 Å.^49^ However, we found that these reactions were not thermodynamically favored, being endergonic by ca. 5 kcal/mol.

**Scheme 2:**
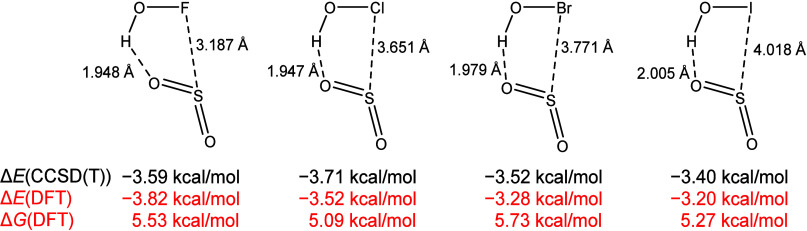
HOX···SO_2_ Interaction Model

In our previous studies, the reactivities of metal-sulfonyl complexes have been revealed, as shown in Scheme 3. In 2024, we investigated the Pd-catalyzed desulfonative Suzuki–Miyaura cross-coupling reaction and found that the Pd-sulfonyl intermediate can undergo a desulfonation step to release a SO_2_ molecule (Scheme 3a).^50^ In 2025, we studied the mechanisms of the competitive metal-catalyzed α-alkylation of sulfones and sulfonyl dissociation, and the conversions of various Mn-sulfonyl intermediates were discovered (Scheme 3b).^51^ Although SO_2_ can be chemically immobilized into organic molecules, achieving efficient and reversible SO_2_ fixations remains challenging.^52^ Herein, we wonder whether SO_2_ could react with metal fluoride complexes to form a new type of M–X–SO_2_ complexes and achieve reversible SO_2_ fixation? What is the nature of the new F–S long bond in the M–X–SO_2_ bonding complexes formed? Inspired by recent silico reaction discoveries,^53–57^ we report our theoretical study on new reactivities of SO_2_.

**Scheme 3:**
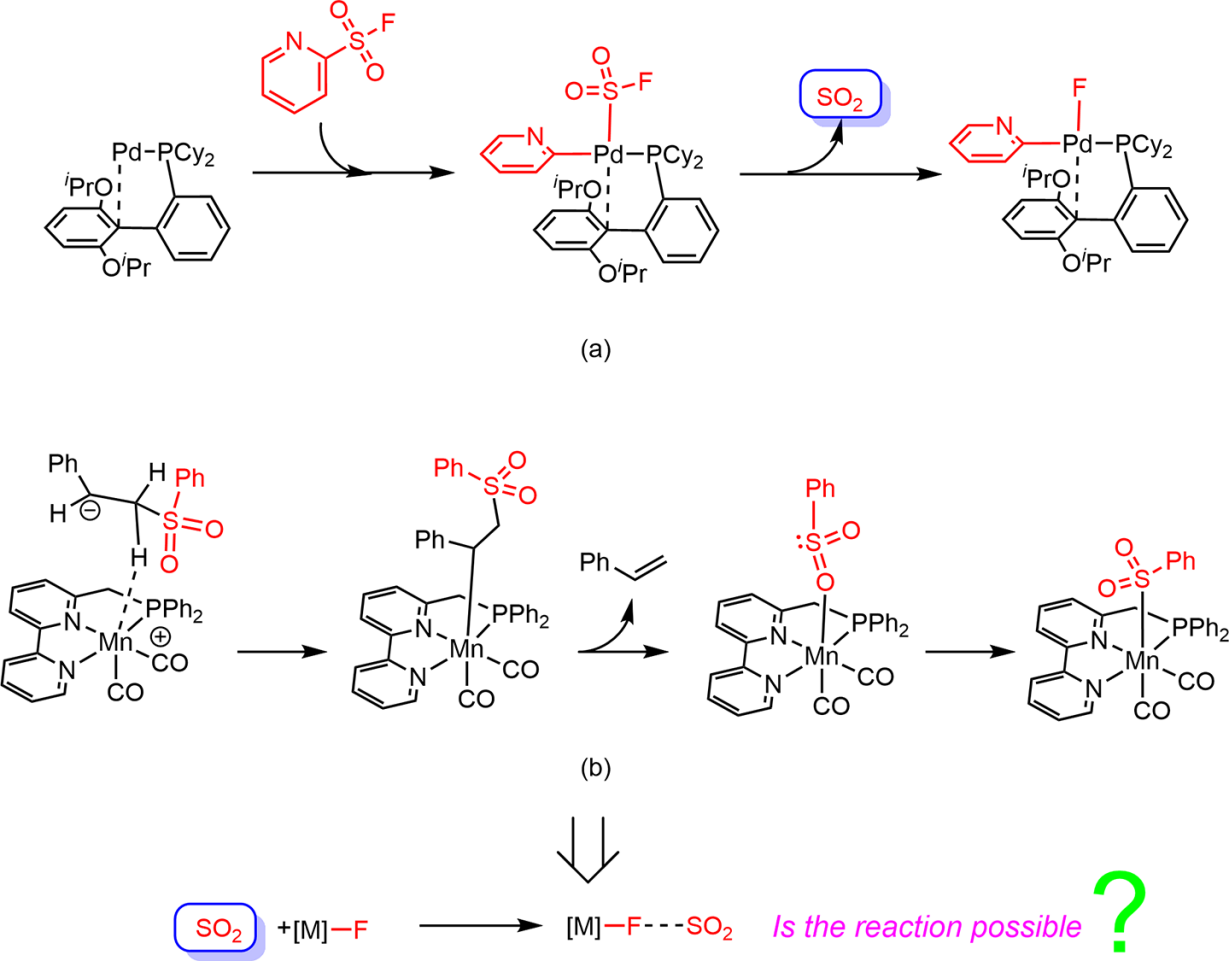
Previous Studies on the Reactivities of Metal-Sulfonyl Complexes in (a) Desulfonative Suzuki–Miyaura Cross-Coupling Reaction; (b) α-Alkylation of Sulfones and Sulfonyl Dissociation

## 2 Computational Methods

In accordance with our previous theoretical studies,^50,58–62^ this research was carried out with the ωB97X-D^63^ method, using the Gaussian 09 program.^64^ Geometries were optimized in 1,4-dioxane solvent using the BS-I basis sets, where the 6-311G­(d, p) basis sets were used for nonmetal atoms and the SDD basis sets with effective core potentials were used for the metal atoms.^65,66^ The single-point energy refinements were further performed with the BS-II basis sets, where the 6-311++G­(2d, p) basis sets were used for nonmetal atoms, and the SDD basis sets with effective core potentials were used for the metal atoms. The refined energies were then corrected to Gibbs energies at standard temperature and pressure (298.15 K and 1 atm) by using the ωB97X-D/BS-I harmonic frequencies. The solvent effect was evaluated using the SMD (solution model based on density) solvation model in both geometry optimization and energy refinement.^67,68^ Harmonic frequency analyses were performed to verify that the optimized geometries were minima. In addition to the ωB97X-D functional, the computations using other functionals are shown in Table S2 (Supporting Information), and all functionals showed the same trend. Natural bond orbital (NBO) analyses were performed using the NBO-7.0 program.^69^ The two-dimensional schematic diagram of the electron density basin and the energy decomposition were determined using the Multiwfn program.^70^ The computed energy results for the highest occupied molecular orbital (HOMO) and the lowest unoccupied molecular orbital (LUMO) are shown in Table S4 (Supporting Information).^71^

## 3 Results and Discussion

### 3.1 New Reactivity of SO_2_

As shown in Figure 1, the Ni–F complex (**1A**) supported by a bidentate PN ligand is used as the acceptor model for reversible SO_2_ absorption. The natural charges of the F(1) and F(2) atoms in **1A** are −0.76 and −0.71, respectively. The SO_2_ molecule can coordinate with the more negative F(1) ligand to form the Ni–F–SO_2_ complex **1B**, and this step is exergonic by 2.2 kcal/mol, although the entropy penalty. In addition, isomer **1C** with the less negative F(2) atom as the acceptor for SO_2_ is less favorable than **1B** by 2.0 kcal/mol. It is noted that the S–F(1) length in **1B** (2.207 Å) is 0.402 Å longer than that of the classical S–F covalent bond (1.805 Å) in the FSO_2_^–^ anion (Figure 2). Therefore, the S–F long bond in **1B** is not a traditional covalent bond. The formation of the S–F long bond elongates the Ni–F(1) bond in **1B** by 0.054 Å compared with that in **1A**. Other possible isomers of **1B** were also considered. The unusual S–F long bond in **1B** could further be rearranged into the classical S–F covalent bonds (1.764 and 1.733 Å) in isomers **1D** and **1F**, respectively. Interestingly, the free energies of **1D** and **1F** are 2.6 and 12.6 kcal/mol higher than that of **1B**. The formation of intermediates **1D**, **1E**, **1F**, and **1G** through the ligand rearrangements of **1B**, **1C** is an energy uphill process. The coordination of SO_2_ with the Ni center to form complexes **1H** is considered, but it is endergonic by 15.3 kcal/mol. In brief, intermediate **1B** is the most stable SO_2_-bounded complex with a binding free energy (Δ*G*_bind_) of −2.2 kcal/mol, representing the reversible SO_2_ absorption in terms of the chemical equilibrium. The Cartesian coordinates of all optimized structures are presented in the Supporting Information.

**Figure 1:**
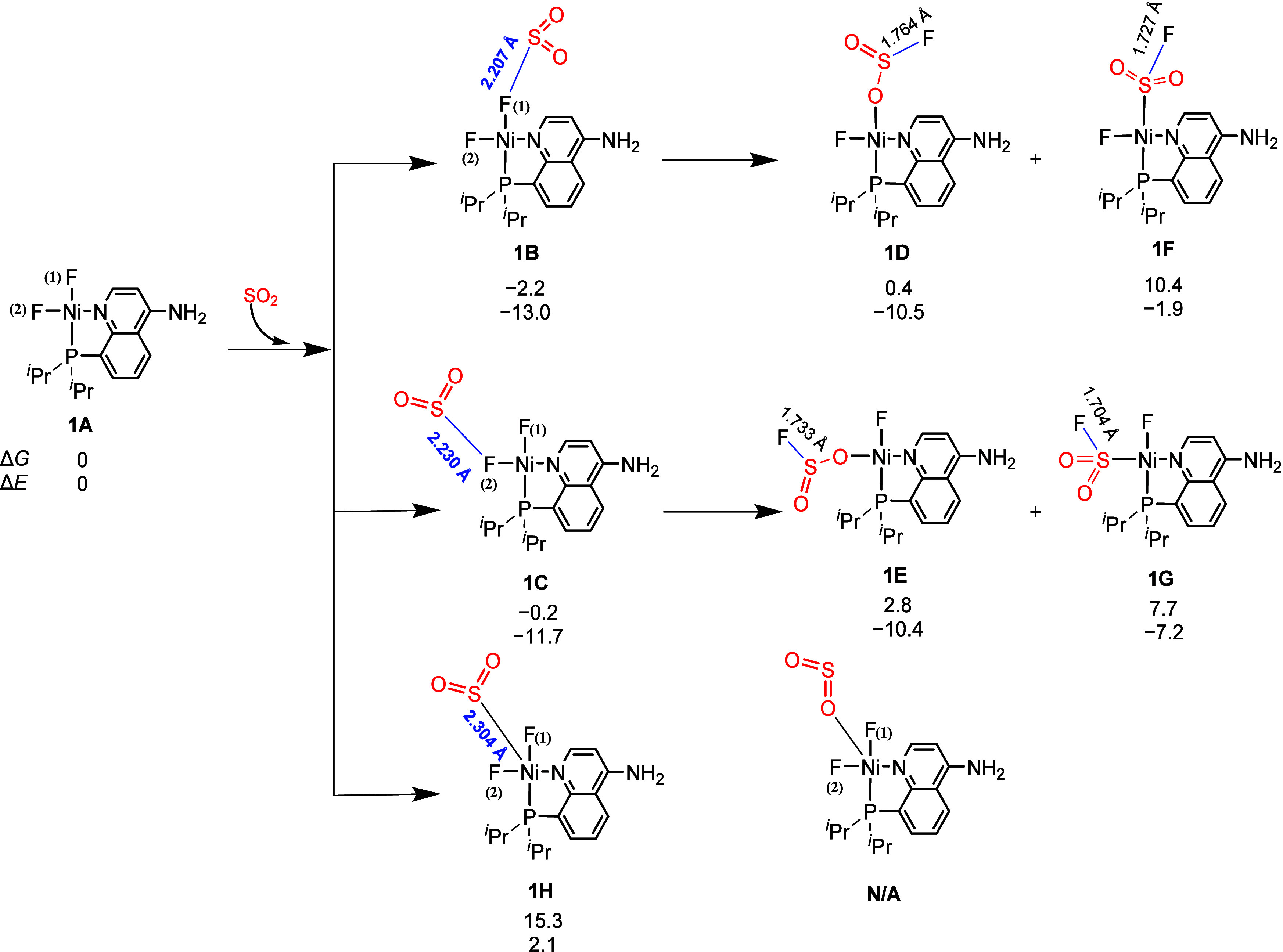
Reversible absorption of SO_2_ by the Ni–F complex through the formation of a new S–F long bond. The relative Gibbs free energies (Δ*G*) and potential energies (Δ*E*) are given in kcal/mol. n/a denotes that the structure is not available.

**Figure 2:**
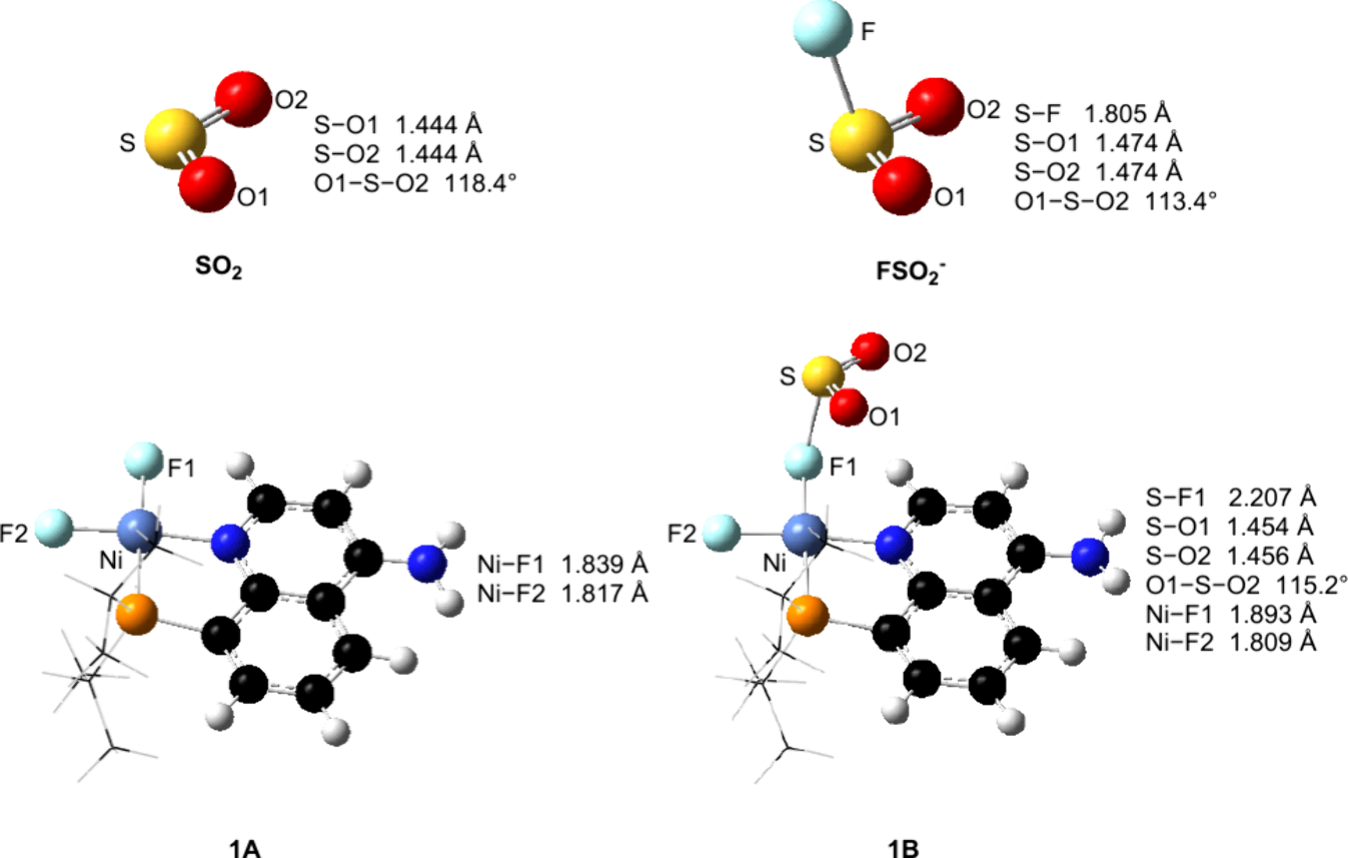
Structural comparisons of SO_2_, FSO_2_^–^ anion, **1A**_**,**_ and **1B.**

### 3.2 Comparison between the New S–F Long Bond and the Classical S–F Bond

To understand the nature of the new S–F long bond, the natural localized molecular orbital (NLMO) analysis was performed on **1B**.^72^ The corresponding second-order perturbative energy *E* ^(2)^ is the energetic stabilization associated with excitations from an occupied Lewis-type natural bond orbital of the formal Lewis structure to an unoccupied non-Lewis-type natural bond orbital.^73^ As shown in Figure 3a, the lone pair electrons of the F(1) atom are donated to the π*­(O=S=O) antibonding orbital, and the corresponding second-order perturbative energy *E* ^(2)^ for the donor–acceptor interaction p­(F) → π*­(O=S=O) is 37.3 kcal/mol. Furthermore, the NLMO of the free FSO_2_^–^ anion is compared with that of the S–F restrained FSO_2_^–^ geometry in order to analyze the reason for p­(F) → π*­(SO_2_) bonding interaction in **1B**. The free FSO_2_^–^ anion with an S–F bond length of 1.805 Å displays the traditional σ­(S–F) bond interaction (Figure 3b). When the S–F length in the FSO_2_^–^ anion is restrained to be the same length (2.207) in **1B**, the S–F bond restrained FSO_2_^–^ geometry exhibits a p­(F) → π*­(O=S=O) bonding orbital (Figure 3c) similar to that in **1B**. It is noted that no significant overlap between the p­(F) and π*­(SO_2_) exists in HOF···SO_2_ that is reported by Kitzmiller and co-workers.^49^ In brief, the comparison proves that the unique p­(F) → π*­(O=S=O) interaction of the new S–F long bond is induced by the elongated S–F bond.

**Figure 3:**
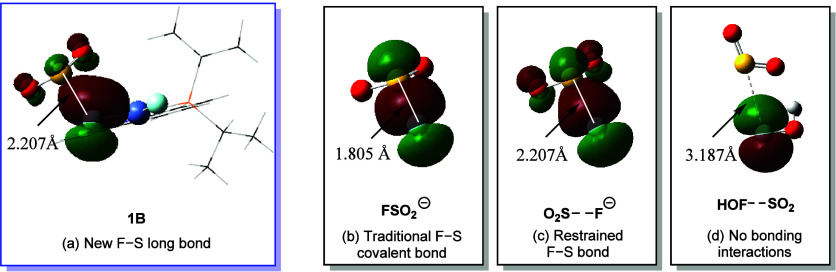
Natural localized molecular orbitals (NLMOs) for the S–F bonding interactions in (a) Ni–F–SO_2_ complex **1B**, (b) free FSO_2_^–^ anion, (c) S–F bond restrained FSO_2_^–^ anion, and (d) HOF···SO_2_.

### 3.3 Comparison between the New S–F Long Bond and Nonbonding Interactions

The new S–F long bond interaction is compared with typical nonbonding interactions, including hydrogen bond and halogen bond interactions. As shown in Figure 4, the Ni–F model complex **1A** bonds the H_2_O or methanol molecule through the hydrogen bond interactions, but the generated hydrogen bond complexes **1H**_**2**_ **O_a** and **1H**_**2**_ **O_b** are less thermodynamically viable than **1B**. In terms of the halogen bond interactions, the CF_4_, CF_3_Cl, CF_3_Br, and CF_3_I are reported to be strong halogen bond donors^74,75^ and selected for the comparisons here. The molecules CF_4_ and **1A** cannot form a halogen bond complex because they cannot be located in the geometry optimization. The CF_3_Cl, CF_3_Br, and CF_3_I molecules are difficult to form halogen bond complexes with **1A** because these processes are endergonic by 8.2, 5.6, and 1.8 kcal/mol, respectively. In brief, the comparison suggests that the strength of new S–F long bond is much stronger than the hydrogen bond and halogen bond interactions.

**Figure 4:**
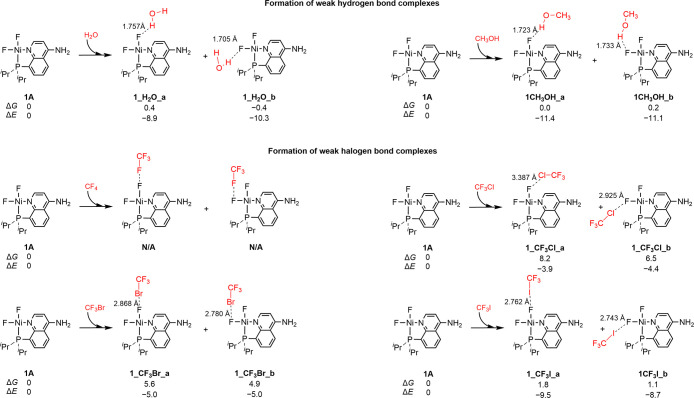
Formation of weak hydrogen bond and halogen bond complexes for structure **1A**. The relative Gibbs free energies (Δ*G*) and potential energies (Δ*E*) are in kcal/mol.

### 3.4 Factors Influencing the Strength of New S–F Long Bonds

The substituents and X ligands in **1B** were varied to investigate the factors influencing the strength of the new S–F long bond. As shown in Figure 5a, the charge of F(1) atoms is more negative than that of F(2) atoms (Figure S2 in SI), and the blue line lies below the red line suggesting that the negativity of F atoms favors high binding free energies (Δ*G*_bind_). Moreover, the electron-donating −OH, −Me, −NH_2_, and −SO_3_K substituents display more negative binding free energies (Δ*G*_bind_) compared with the –H substituent. In contrast, the electron-withdrawing −CHO, −CN, −NO_2_, and −CF_3_ substituents show less negative Δ*G*_bind_ values compared with those of the −H substituent. Therefore, the electronegativity of the substituents is the most important factor for the strength of the new S–F long bond. It should be noted that the electron-donating −^*i*^Pr and −^*t*^Bu have less negative Δ*G*_bind_ values compared with the −H substituent due to the steric resistance.

**Figure 5:**
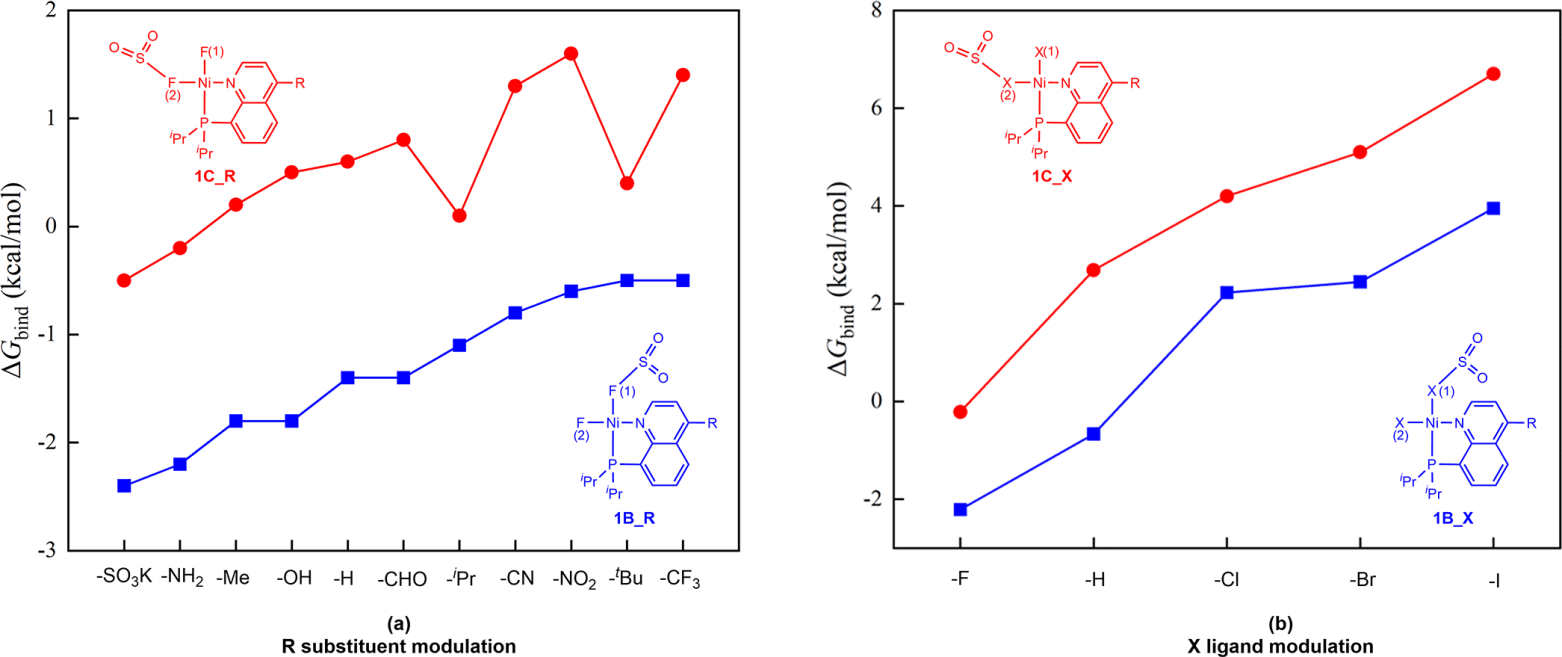
Modulation of (a) R substituents and (b) X ligands in **1B** and **1C** for the formation of a new S–F long bond.

In addition, we also compared the −F ligand of **1A** with the −H, −Cl, −Br, and −I ligands, and the results are illustrated in Figure 5b. The −Cl, −Br, and −I ligands have positive Δ*G*_bind_ values (2.2, 2.4, and 3.9 kcal/mol), indicating that the −Cl, −Br, and −I ligands cannot coordinate with SO_2_ molecules or form the S–X long bond. Interestingly, it is found that the hydride ligand can coordinate with the SO_2_ molecule through a new S–H long bond, and the process is exergonic by 0.7 kcal/mol.

### 3.5 Scope of Stable M–X–SO_2_ Complexes and New S–X Long Bonds

The scope of M–X complexes for the formation of stable M–X–SO_2_ is investigated herein. As shown in Figure 6, various M–X complexes with typical ligands including bisphosphine (**2A**, **3A**),^76,77^ *P,N*-heterobidentate (**4A**),^78^ monodentate (**5A**),^50^ *N,N*-heterobidentate (**6A**),^79^ and pincer (**7A**, **8A**, **9A**, **10A**) ligands are selected.^80–83^ The computations suggest that these M–F complexes can form stable M–X–SO_2_ complexes, and the predicted *G*_bind_ ranges from −1.9 to −0.8 kcal/mol. Interestingly, the anionic M–F complexes **10A** and **11A** ^84^ can form much stronger S–F long bonds, being exergonic by 7.8 and 9.5 kcal/mol, respectively. Furthermore, the anionic property can endow M–H and M–Cl complexes (**12A**, **13A**)^84^ with the ability to form stable M–X–SO_2_ complexes and S–X long bonds. More M–F complexes for forming stable M–X–SO_2_ complexes with SO_2_ are shown in Figures S12–S20 of SI. In brief, the study indicates the universality of this S–X long bond and that anionic properties can significantly enhance this kind of bond.

**Figure 6:**
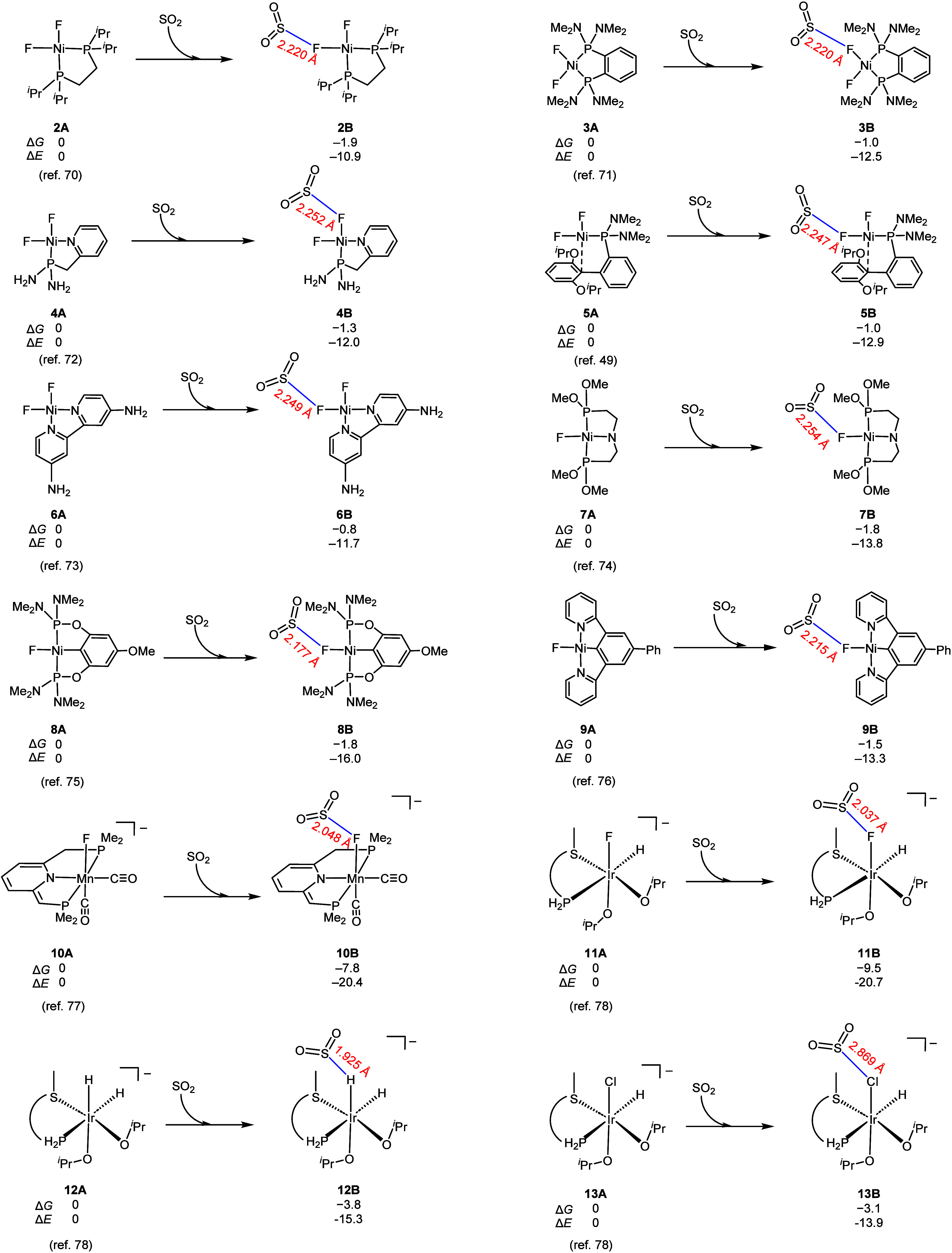
M–X complexes (X = F, Cl, H) with different ligand skeletons for the formation of stable M–X–SO_2_ complexes. The relative Gibbs free energies (Δ*G*) and potential energies (Δ*E*) are in kcal/mol.

## 4 Conclusions

This study elucidates new reactivates of SO_2_ which could be applied in a reversible SO_2_ absorption process. The new Ni–F–SO_2_ complex **1B** is found to be the most stable SO_2_-bound complex with a binding free energy (Δ*G*_bind_) of −2.2 kcal/mol, compared to other conventional O-bonded (**1D**) and S-bonded (**1F**) metal-sulfonyl complexes. The long S–F bond displays a length of 2.207 Å and is much longer than conventional S–F covalent bonds. NLMO analysis reveals that the long S–F bond features a p­(F) → π*­(O=S=O) donor–acceptor interaction, which is induced by the elongation of the S–F bond. The comparison suggests that the Ni–F–SO_2_ complex **1B** is much more stable than classical hydrogen bond and halogen bond complexes, and the new S–F long bond is significantly stronger than hydrogen and halogen nonbonding interactions.

The substituents suggest that electron-donating groups (−OH, −Me, −NH_2_, −SO_3_K) significantly enhance the binding affinity compared to the −H substituent, as reflected by a more negative binding free energy (Δ*G*_bind_). In contrast, electron-withdrawing groups (−CHO, −CN, −NO_2_, and −CF_3_) weaken the binding interaction. The scope of M–X complexes for the formation of stable M–X–SO_2_ complexes is investigated. The M–F complexes bearing various ligands were found to bind SO_2_ via the S–F long interactions, with predicted binding free energies (Δ*G*_bind_) ranging from −1.9 to −0.8 kcal/mol. Anionic M–F complexes (**10A**, **11A**) exhibit much stronger binding affinities with higher Δ*G*_bind_ values (−7.8 and −9.5 kcal/mol). Interestingly, their anionic character can allow M–H (**12A**) and M–Cl (**13A**) complexes to form long S–H and S–Cl long bonds. These results prove that the S–X long bonds can widely exist in M–X complexes, and the anionic strategy can significantly enhance the bonding strength. This study will pave the way for a green, recyclable, and adjustable SO_2_ absorption method.

## Supplementary Material





## Notes

Optimized energies of all stationary points; comparison of different DFT functionals; comparison of different solvents; the electron density basin; the energy decomposition; other M–F complexes for forming stable M–X–SO_2_ complexes (PDF)
Coordinates of the optimized structures (XYZ)
