# Supplementary material for: Unveiling Unusual Reactivity of SO2 and Unusual Type of S–X Long Bonds
Source: Inorg Chem. 2025 Jul 8;64(28):14684–92. doi: 10.1021/acs.inorgchem.5c02435 (PMC12284863; doi:10.1021/acs.inorgchem.5c02435)
Supplement: Supplementary file 1 [file ic5c02435_si_001.pdf]

Supporting Information

Unveiling Unusual Reactivity of SO_2 and Unusual Type of S−X Long Bonds

Shuai Ma,[§] Longfei Li,[a]* Xiaofeng Xie,[§] Peng Wang,[a] He Bai,[a] Kan Yang,[a] Xueqing Song,[a] Henry F. Schaefer III[b]*

[a] *College of Pharmacy, Key Laboratory of Pharmaceutical Quality Control of Hebei Province, Key Laboratory of Medicinal Chemistry and Molecular Diagnosis of Ministry of Education, Hebei University, Baoding 071002, Hebei, P. R. China*

[b] Center for Computational Quantum Chemistry, University of Georgia, Athens, Georgia 30602, USA.

*Email: lilongfei@hbu.edu.cn (L.L.); ccq@uga.edu (H.S.)

Table S1. The computed potential energies (E, a.u.) and free energies (ΔG; at 298.15 K) are in kcal/mol at the ωB97X-D/BS-II level.........S3
Figure S1. The unfavorable coordination of SO_2 with the Ni center in **1A**. S8
Table S2. The comparison of different DFT functionals for the computed potential energies and relative potential energies of **1B**. The energies are in kcal/mol.........S9
Table S3. The comparison of different solvents for the computed potential energies and relative potential energies of **1B**. The energies are in kcal/mol. S9
Table S4. The HOMO, LUMO, ionization energy, electron affinity for the computed potential energies and relative potential energies of **1A**. S10
Table S5. The energy decomposition results for the computed potential energies and relative potential energies of **1B** are in kcal/mol. S11
Figure S2. Molecular Electrostatic Potential (MEP) surfaces for the **1A**. S12
Figure S3. The two-dimensional schematic of the electron density basin for **1B**. S13
Figure S4. The structure and NBO charges of **1A**.........S14
Figure S5. The relaxed potential energies surface scan along the S···F distance for the anion FSO_2^-. S15
Figure S6. The natural localized molecular orbitals (NLMOs) analysis on the F···H and F···Cl interactions in **1_H₂O_a** and **1_CF₃Cl_a**.........S16
Figure S7. The formation of halogen bond complexes between the structure **1A** and other halogen bond donors. The relative Gibbs free energies (ΔG) and potential energies (ΔE) are in kcal/mol. S17

Figure S8. The formation of chalcogen bond complexes between the structure **1M**, **1N** and SO_2. The relative Gibbs free energies (ΔG) and potential energies (ΔE) are in kcal/mol....................................S18
Figure S9. The formation of hydrogen bond complexes between SO_2 and glycine **1O**. The relative Gibbs free energies (ΔG) and potential energies (ΔE) are in kcal/mol. S19
Figure S10. Modeling the bidentate coordination of SO_2 with two fluoride atoms. The relative Gibbs free energies (ΔG) and potential energies (ΔE) are in kcal/mol. S20
Figure S11. The nitrogen atom of the -NH_2 group as absorption sites. The relative Gibbs free energies (ΔG) and potential energies (ΔE) are in kcal/mol. S21
Figure S12. The absorption of SO_2 by the bisphosphine Ni complexes **2A** and **3A** with different substituents. The relative Gibbs free energies (ΔG) and potential energies (ΔE) are in kcal/mol.........S22
Figure S13. The absorption of SO_2 by the bisphosphine Ni complexes **14A** with different X ligands. The relative Gibbs free energies (ΔG) and potential energies (ΔE) are in kcal/mol....................................S23
Figure S14. The absorption of SO_2 by the *P*,*N*-heterobidentate Ni complexes **4A** and **15A** with different substituents. The relative Gibbs free energies (ΔG) and potential energies (ΔE) are in kcal/mol.........S24
Figure S15. The absorption of SO_2 by the *P*,*N*-heterobidentate ligand Ni complexes **16A** with different substituents. The relative Gibbs free energies (ΔG) and potential energies (ΔE) are in kcal/mol.........S25
Figure S16. The absorption of SO_2 by the monodentate ligand Ni complexes **5A** with different substituents. The relative Gibbs free energies (ΔG) and potential energies (ΔE) are in kcal/mol.............................S26
Figure S17. The absorption of SO_2 by the *N*,*N*-heterobidentate ligand Ni complexes **6A** and **17A** with different substituents. S27
Figure S18. The absorption of SO_2 by the pincer ligand Ni complexes **8A** and **18A** with different substituents. The relative Gibbs free energies (ΔG) and potential energies (ΔE) are in kcal/mol.........S28
Figure S19. The absorption of SO_2 by the pincer ligand Ni complexes **9A** with different substituents. The relative Gibbs free energies (ΔG) and potential energies (ΔE) are in kcal/mol....................................S29
Figure S20. The absorption of SO_2 by the pincer ligand Ni complexes **19A** with different substituents. The relative Gibbs free energies (ΔG) and potential energies (ΔE) are in kcal/mol.............................S30
Figure S21. The comparisons of the Pd and Pt metals for the absorption of SO_2 by complex **1A**. The relative Gibbs free energies (ΔG) and potential energies (ΔE) are in kcal/mol....................................S31
Figure S22. The calculation of the conversion rate for the formation of the complex **1B**....................S32

Table S1. The computed potential energies (E, a.u.) and free energies (ΔG; at 298.15 K) are in kcal/mol at the ωB97X-D/BS-II level.

Complex	E	ΔE	G	ΔG
**1A**	-1405.8714	0.0	-1954.2253	0
**1B**	-1954.5189	-13.0	-1954.2253	-2.2
**1C**	-1954.5167	-11.7	-1954.2222	-0.2
**1D**	-1954.5149	-10.5	-1954.2212	0.4
**1E**	-1954.5148	-10.4	-1954.2173	2.8
**1F**	-1954.5011	-1.9	-1954.2052	10.4
**1G**	-1954.5097	-7.2	-1954.2096	7.7
**1H**	-1954.4948	2.1	-1954.1974	15.3
**1_H_2O_a**	-1482.3258	-10.4	-1482.0112	0.4
**1_H_2O_b**	-1482.3256	-10.3	-1482.0125	-0.4
**1_CH_2OH_a**	-1521.6182	-11.7	-1058.2468	0.0
**1_CH_2OH_b**	-1521.6173	-11.1	-1521.2772	0.2
**1_CF_3Cl_a**	-2203.7385	-3.9	-2203.4387	8.2
**1_CF_3Cl_b**	-2203.7392	-4.4	-2203.4414	6.5
**1_CF_3Br_a**	-1756.9102	-5.0	-1756.6148	5.6
**1_CF_3Br_b**	-1756.9102	-5.0	-1756.6159	4.9
**1_CF_3I_a**	-1754.9517	-9.5	-1754.6567	1.8
**1_CF_3I_b**	-1754.9504	-8.7	-1754.6577	1.1
**1A_SO_3K**	-2573.7767	0.0	-2573.5040	0.0
**1A_Me**	-1389.8212	0.0	-1389.5177	0.0
**1A_OH**	-1425.7355	0.0	-1425.4547	0.0
**1A_H**	-1350.5007	0.0	-1350.2234	0.0
**1A_CHO**	-1463.8173	0.0	-1463.5334	0.0
**1A_^{i}Pr**	-1468.4482	0.0	-1468.0900	0.0
**1A_CN**	-1442.7264	0.0	-1442.4538	0.0
**1A_NO_2**	-1554.9907	0.0	-1554.7148	0.0
**1A_^{t}Bu**	-1507.7553	0.0	-1507.3705	0.0
**1A_CF_3**	-1687.5600	0.0	-1687.2833	0.0
**1B_SO_3K**	-3122.4248	-13.3	-3122.1517	-2.4
**1B_Me**	-1938.4680	-12.7	-1938.1643	-1.8
**1B_OH**	-1974.3826	-12.6	-1974.1014	-1.8
**1B_H**	-1899.1475	-12.6	-1898.8694	-1.4
**1B_CHO**	-2012.4636	-12.3	-2012.1794	-1.4
**1B_^{i}Pr**	-2017.0951	-12.6	-2016.7356	-1.1
**1B_CN**	-1991.3723	-12.0	-1991.0988	-0.8
**1B_NO_2**	-2103.6366	-12.0	-2103.3595	-0.6
**1B_^{t}Bu**	-2056.4023	-12.7	-2056.0150	-0.5
**1B_CF_3**	-2236.2061	-12.1	-2235.9279	-0.5
**1C_SO_3K**	-3122.4212	-11.1	-3122.1487	-0.5
**1C_Me**	-1938.4662	-11.4	-1938.1613	0.2

**1C_OH**	-1974.3799	-11.0	-1974.0976	0.5
**1C_H**	-1899.1454	-11.2	-1898.8663	0.6
**1C_CHO**	-2012.4613	-10.8	-2012.1759	0.8
**1C_^{i}Pr**	-2017.0932	-11.4	-2016.7337	0.1
**1C_CN**	-1991.3702	-10.7	-1991.0954	1.3
**1C_NO_2**	-2103.6347	-10.8	-2103.3560	1.6
**1C_^{t}Bu**	-2056.4002	-11.4	-2056.0136	0.4
**1C_CF_3**	-2236.2043	-11.0	-2235.9248	1.4
**1A_X_H**	-1207.2598	0.0	-1206.9552	0.0
**1A_X_Cl**	-2126.6342	0.0	-2126.3449	0.0
**1A_X_Br**	-1232.9861	0.0	-1232.6990	0.0
**1A_X_I**	-1229.0615	0.0	-1228.7766	0.0
**1B_X_H**	-1755.9073	-13.0	-1755.6000	-0.7
**1B_X_Cl**	-2675.2737	-8.0	-2674.9850	2.3
**1B_X_Br**	-1781.6254	-7.9	-1781.3389	2.4
**1B_X_I**	-1777.6995	-7.1	-1777.4141	3.9
**1C_X_H**	-1755.9013	-9.2	-1755.5947	2.7
**1C_X_Cl**	-2675.2722	-7.0	-2674.9820	4.2
**1C_X_Br**	-1781.6221	-5.8	-1781.3347	5.1
**1C_X_I**	-1777.6961	-4.9	-1777.4096	6.7
**2A**	-1606.2860	0.0	-1605.8908	0.0
**3A**	-1822.8216	0.0	-1822.4523	0.0
**4A**	-1111.0386	0.0	-1110.9092	0.0
**5A**	-1830.2109	0.0	-1829.7457	0.0
**6A**	-976.8404	0.0	-976.6836	0.0
**7A**	-1626.1459	0.0	-1625.8986	0.0
**8A**	-1987.3489	0.0	-1986.9611	0.0
**9A**	-1227.6923	0.0	-1227.4249	0.0
**10A**	-1598.5238	0.0	-1598.2861	0.0
**11A**	-1489.9100	0.0	-1489.6316	0.0
**12A**	-1499.2165	0.0	-1498.9725	0.0
**13A**	-1390.6359	0.0	-1390.3515	0.0
**2B**	-2154.9302	-10.9	-2154.5375	-1.9
**3B**	-2371.4683	-12.5	-2371.0978	-1.0
**4B**	-1659.6844	-12.0	-1659.5550	-1.3
**5B**	-2378.8582	-12.9	-2378.3911	-1.0
**6B**	-1525.4858	-11.7	-1525.3287	-0.8
**7B**	-2174.7946	-13.8	-2174.5453	-1.8
**8B**	-2536.0011	-16.0	-2535.6077	-1.8
**9B**	-1776.3402	-13.3	-1776.0711	-1.5
**10B**	-2147.1830	-20.4	-2146.9423	-7.8
**11B**	-2038.5699	-20.8	-2038.2904	-9.5
**12B**	-2047.8792	-22.6	-2047.6310	-9.2
**13B**	-1939.2870	-15.3	-1939.0013	-3.8
**1I**	-1494.0204	-9.4	-1493.7185	0.1

**1J**	-1494.0199	-9.1	-1493.7189	-0.2
**1K**	-2056.6971	-9.5	-2056.3765	2.3
**1L**	-2056.6962	-8.9	-2056.3773	1.9
**2A_C_6F_5**	-3944.1106	0.0	-3943.9151	0.0
**2B_C_6F_5**	-4492.7556	-11.5	-4492.5610	-1.3
**2A_^{t}Bu**	-1763.5304	0.0	-1763.0237	0.0
**2B_^{t}Bu**	-2312.1772	-12.6	-2311.6686	-0.7
**2A_Me**	-1291.7748	0.0	-1291.6013	0.0
**2B_Me**	-1840.4219	-12.8	-1840.2457	-0.4
**2A_NMe**	-1670.4110	0.0	-1670.0629	0.0
**2B_NMe**	-2219.0544	-10.4	-2218.7070	-0.2
**3A_^{t}Bu**	-1915.9338	0.0	-1915.4064	0.0
**3B_^{t}Bu**	-2464.5810	-12.8	-2464.0512	-0.6
**3A_Ph**	-2211.0486	0.0	-2210.6559	0.0
**3B_Ph**	-2759.6943	-11.8	-2759.3006	-0.5
**14A_PhOMe**	-1852.5456	0.0	-1852.0377	0.0
**14B_PhOMe**	-2401.1959	-14.7	-2400.6855	-2.5
**14A_Me**	-1546.2985	0.0	-1545.8710	0.0
**14B_Me**	-2094.9461	-13.0	-2094.5187	-2.5
**14A_CF_3**	-1844.0666	0.0	-1843.6637	0.0
**14B_CF_3**	-2392.7144	-13.2	-2392.3107	-2.0
**14A_$PhNH_2$**	-1793.3825	0.0	-1792.8888	0.0
**14B_$PhNH_2$**	-2342.0332	-15.1	-2341.5356	-1.9
**14A_Ph**	-1738.0234	0.0	-1737.5473	0.0
**14B_Ph**	-2286.6728	-14.2	-2286.1926	-0.9
**14A_C_2F_3**	-1882.1114	0.0	-1881.7057	0.0
**14B_C_2F_3**	-2430.7567	-11.6	-2430.3507	-0.8
**4A_NH_2**	-1111.0386	0.0	-1110.9092	0.0
**4B_NH_2**	-1659.6844	-12.0	-1659.5550	-1.3
**4A_Ph**	-1462.3643	0.0	-1462.1150	0.0
**4B_Ph**	-2011.0101	-11.9	-2010.7604	-1.0
**4A_^{i}Pr**	-1236.1870	0.0	-1235.9274	0.0
**4B_^{i}Pr**	-1784.8335	-12.4	-1784.5726	-0.9
**4A_^{t}Bu**	-1314.8157	0.0	-1314.5003	0.0
**4B_^{t}Bu**	-1863.4626	-12.6	-1863.1454	-0.8
**4A_Me**	-1078.9307	0.0	-1078.7813	0.0
**4B_Me**	-1627.5773	-12.4	-1627.4255	-0.3
**4A_OMe**	-1229.3696	0.0	-1229.2115	0.0
**4B_OMe**	-1778.0157	-12.2	-1777.8556	-0.2
**15A_OMe**	-1350.7174	0.0	-1350.4278	0.0
**15B_OMe**	-1899.3643	-12.7	-1899.0737	-1.3
**15A_^{t}Bu**	-1393.4497	0.0	-1393.0833	0.0
**15B_^{t}Bu**	-1942.0963	-12.5	-1941.7290	-1.2
**15A_OH**	-1311.4217	0.0	-1311.1587	0.0
**15B_OH**	-1860.0685	-12.6	-1859.8035	-0.7

**15A_F**	-1335.4309	0.0	-1335.1810	0.0
**15B_F**	-1884.0771	-12.2	-1883.8254	-0.4
**15A_NMe**	-1370.1658	0.0	-1369.8380	0.0
**15B_NMe**	-1918.8130	-12.8	-1918.4823	-0.4
**16A_NH$_2$**	-1264.6693	0.0	-1264.4972	0.0
**16B_NH$_2$**	-1813.3145	-11.6	-1813.1426	-1.0
**16A_*i*Pr**	-1389.8201	0.0	-1389.5166	0.0
**16B_*i*Pr**	-1938.4669	-12.6	-1938.1615	-0.7
**16A_*t*Bu**	-1468.4480	0.0	-1468.0906	0.0
**16B_*t*Bu**	-2017.0950	-12.7	-2016.7353	-0.6
**16A_Me**	-1232.5651	0.0	-1232.3723	0.0
**16B_Me**	-1781.2112	-12.1	-1781.0168	-0.5
**16A_OH**	-1304.4292	0.0	-1304.2804	0.0
**16B_OH**	-1853.0742	-11.4	-1852.9246	-0.3
**16A_H**	-1153.9061	0.0	-1153.7666	0.0
**16B_H**	-1702.5503	-10.9	-1702.4107	-0.2
**5A_*i*Pr**	-1798.1494	0.0	-1797.6623	0.0
**5B_*i*Pr**	-2346.7965	-12.8	-2346.3072	-0.7
**5A_Et**	-1719.5192	0.0	-1719.0860	0.0
**5B_Et**	-2268.1668	-13.0	-2267.7308	-0.6
**5A_Me**	-1640.8959	0.0	-1640.5193	0.0
**5B_Me**	-2189.5428	-12.6	-2189.1638	-0.5
**5A_Ph**	-2024.3302	0.0	-2023.8521	0.0
**5B_Ph**	-2572.9769	-12.5	-2572.4964	-0.3
**6A_*t*Bu**	-1180.6241	0.0	-1180.2833	0.0
**6B_*t*Bu**	-1729.2689	-11.3	-1728.9279	-0.5
**17A_Me**	-868.4661	0.0	-868.2998	0.0
**17B_Me**	-1417.1122	-12.1	-1416.9448	-0.7
**8A_NH$_2$**	-1672.9190	0.0	-1672.7459	0.0
**8B_NH$_2$**	-2221.5744	-18.0	-2221.3937	-2.5
**8A_C$_6$F$_5$**	-4360.2662	0.0	-4360.0369	0.0
**8B_C$_6$F$_5$**	-4908.9147	-13.7	-4908.6820	-0.8
**18A_NH$_2$**	-1558.3947	0.0	-1558.2503	0.0
**18B_NH$_2$**	-2107.0499	-17.8	-2106.8982	-2.6
**18A_NMe**	-1872.8247	0.0	-1872.4657	0.0
**18B_NMe**	-2421.4722	-13.0	-2421.1097	-0.1
**9A_NH$_2$**	-1052.0148	0.0	-1051.8075	0.0
**9B_NH$_2$**	-1600.6604	-11.8	-1600.4525	-0.7
**9A_OH**	-1071.8825	0.0	-1071.6876	0.0
**9B_OH**	-1620.5278	-11.6	-1620.3322	-0.6
**9A_Me**	-1035.9718	0.0	-1035.7553	0.0
**9B_Me**	-1584.6172	-11.6	-1584.3998	-0.4
**9A_*i*Pr**	-1114.6006	0.0	-1114.3296	0.0
**9B_*i*Pr**	-1663.2461	-11.8	-1662.9736	-0.1
**19A_NMe**	-1165.0701	0.0	-1164.7906	0.0

**19B_NMe**	-1713.7164	-12.3	-1713.4356	-0.8
**19A_Me**	-1070.4222	0.0	-1070.1867	0.0
**19B_Me**	-1619.0679	-11.9	-1618.8312	-0.5
**19A_OMe**	-1145.6278	0.0	-1145.3875	0.0
**19B_OMe**	-1694.2735	-11.9	-1694.0319	-0.4
**19A_NH2**	-1086.4652	0.0	-1086.2391	0.0
**19B_NH2**	-1635.1113	-12.1	-1634.8832	-0.2
**1A_Pd**	-1362.8336	0.0	-1362.5412	0.0
**1B_Pd**	-1911.4825	-13.8	-1911.1888	-2.4
**1C_Pd**	-1911.4807	-12.7	-1911.1851	-0.1
**1A_Pt**	-1354.3412	0.0	-1354.0502	0.0
**1B_Pt**	-1902.9882	-12.7	-1902.6952	-0.8
**1C_Pt**	-1902.9874	-12.2	-1902.6916	1.5

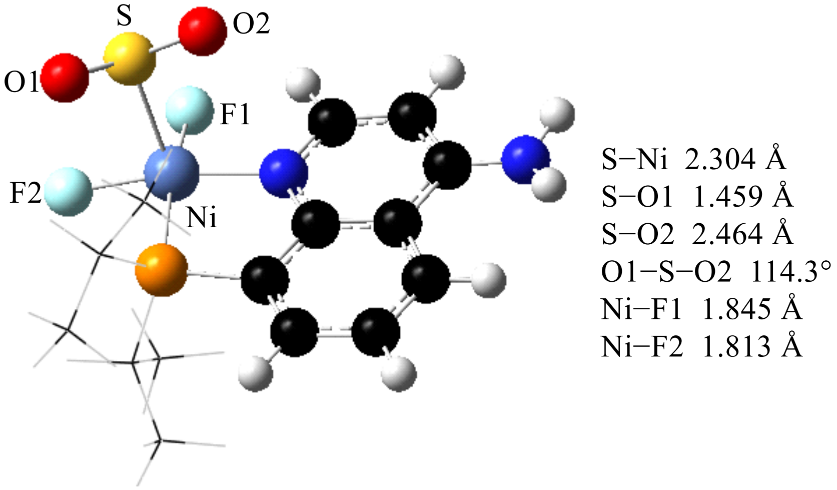

Figure S1. The unfavorable coordination of SO_2 with the Ni center in **1A**.

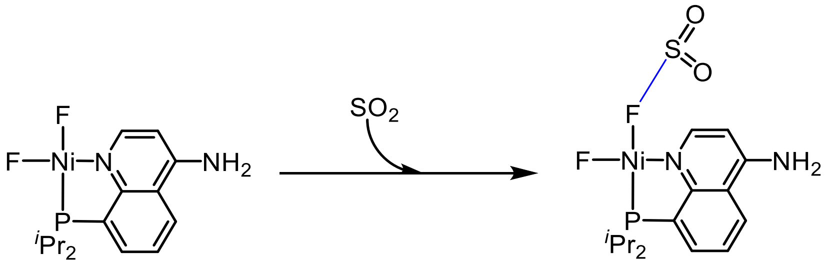

1A **1B**

Δ*G*

Δ*E*

Table S2. The comparison of different DFT functionals for the computed potential energies and relative potential energies of **1B**. The energies are in kcal/mol.

Methods	Δ*E*	Δ*G*
ωB97X-D/SDD/6-311++G(2d, p)//ωB97X-D/SDD/6-311G(d, p)	-13.0	-2.2
ωB97X-D/SDD/6-311+G(d, p)//ωB97X-D/SDD/6-311G(d, p)	-14.4	-3.6
B3LYP-D3/SDD/6-311++G(2d, p)//B3LYP-D3/SDD/6-311G(d, p)	-15.3	-3.9
B3PW91-D3/SDD/6-311++G(2d, p)//B3PW91-D3/SDD/6-311G(d, p)	-15.7	-3.7
MN15/SDD/6-311++G(2d, p)//MN15/SDD/6-311G(d, p)	-16.3	-4.1
ωB97X-D/def2-TZVP	-13.2	-1.9
ωB97X-D/cc-pVTZ	-13.9	-3.1

Table S3. The comparison of different solvents for the computed potential energies and relative potential energies of **1B**. The energies are in kcal/mol.

Solvents	Dielectric constant	Δ*E*	Δ*G*
1,4-dioxane	2.2	-13.0	-2.2
Toluene	2.4	-12.6	-1.3
Acetic acid	2.6	-15.1	-2.7
Dichloromethane	8.9	-10.1	2.7
Dimethyl sulfoxide	46.8	-8.3	4.7

Table S4. The HOMO, LUMO, ionization energy, electron affinity for the computed potential energies and relative potential energies of **1A**.

**1A**	Energy
HOMO	-0.29919 ev
LUMO	-0.01513 ev
Ionization energy	156.1 kcal/mol
Electron affinity	-42.4 kcal/mol

**1A_OH**	Energy
HOMO	-0.31036 ev
LUMO	-0.01745 ev
Ionization energy	154.2 kcal/mol
Electron affinity	-44.6 kcal/mol

**1A_H**	Energy
HOMO	-0.31595 ev
LUMO	-0.02725 ev
Ionization energy	156.0 kcal/mol
Electron affinity	-58.3 kcal/mol

**1A_CN**	Energy
HOMO	-0.32206 ev
LUMO	-0.05447 ev
Ionization energy	162.5 kcal/mol
Electron affinity	-65.9 kcal/mol

**1A_CF$_3$**	Energy
HOMO	-0.32215 ev
LUMO	-0.04315 ev
Ionization energy	161.4 kcal/mol
Electron affinity	-59.3 kcal/mol

Table S5. The energy decomposition results for the computed potential energies and relative potential energies of **1B** are in kcal/mol.

Complex	BE	E_{elst}	E_{exch}	E_{ind}	E_{dis}
**1B**	-28.11	-30.84	46.67	-33.37	-13.56

The BE can be decomposed into various physically meaningful components and can be expressed as BE = E_{elst} + E_{exch} + E_{ind} + E_{dis}. The E_{elst} term reflects the classical electrostatic interaction between the monomers. The E_{exch} term denotes the exchange-repulsion contribution caused by the overlap of monomer wave function as well as the antisymmetric requirements due to the fermionic behavior of the electrons in the dimer. The E_{ind} term portrays the induction contribution, which comprises polarization as the response of each monomer to the electric field of the other one as well as charge transfer between two monomers. Finally, the E_{dis} term is the dispersion contribution due to the Coulomb correlation between electrons in one monomer with those in another one.

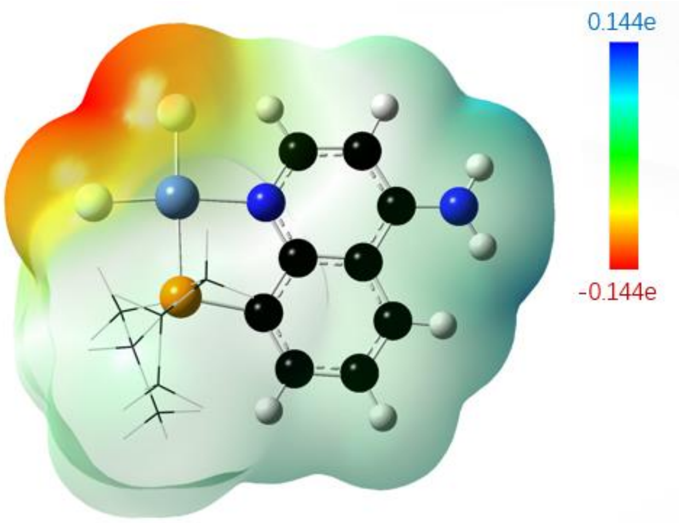

Figure S2. Molecular Electrostatic Potential (MEP) surfaces for the **1A**.

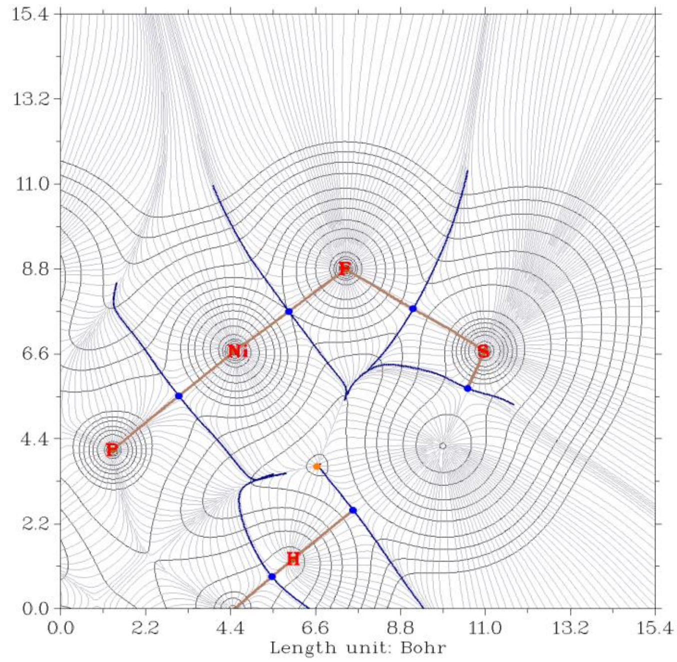

Figure S3. The two-dimensional schematic of the electron density basin for **1B**.

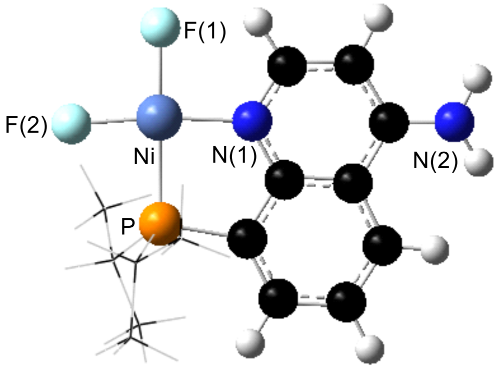

Charge		Lengths
F(1)	−0.757	Ni−F(1) 1.839 Å
F(2)	−0.714	Ni−F(2) 1.817 Å
Ni	0.963	Ni−P 2.165 Å
P	0.983	Ni−N(1) 1.925 Å
N(1)	−0.578	
N(2)	−0.762	

Figure S4. The structure and NBO charges of **1A**.

The structure and NBO charges of **1A** are shown in Figure S6. In structure **1A**, the NBO charges for F(1), F(2), Ni, P, N(1), and N(2) are -0.757, -0.714, +0.963, +0.983, -0.758, and **-**0.762, respectively. In structure **1A**, the lengths of Ni−F(1), Ni−F(2), Ni−P and Ni−N(1) are 1.839, 1.817, 2.165 and 1.925 Å, respectively.

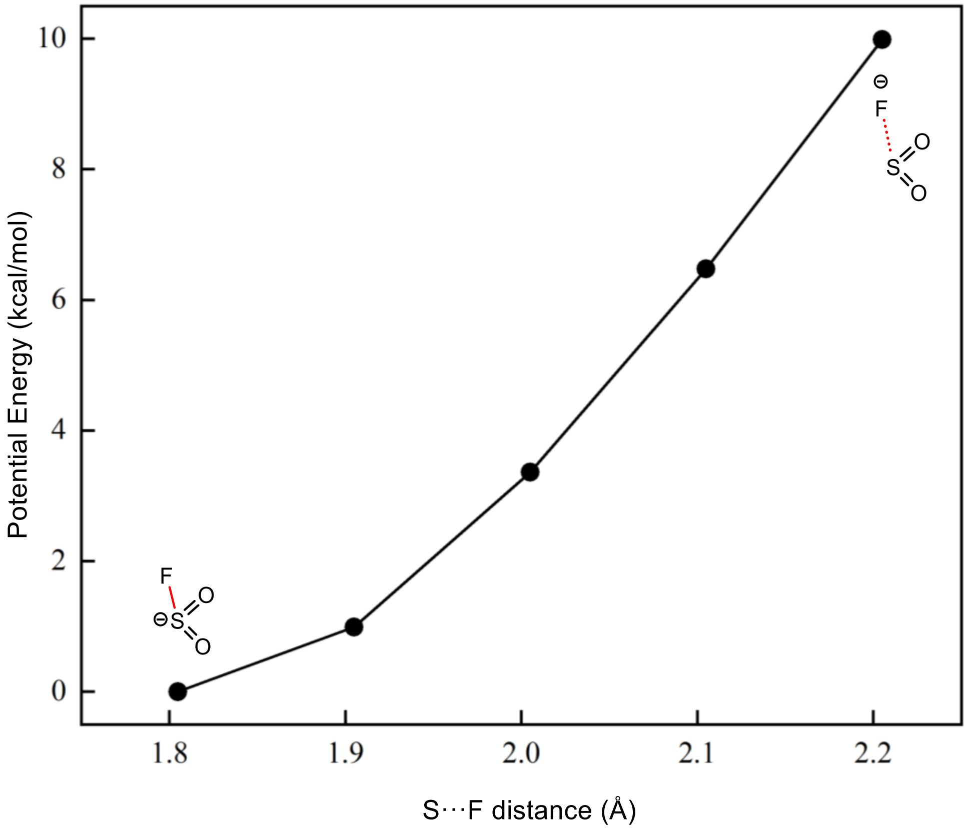

Figure S5. The relaxed potential energies surface scan along the S···F distance for the anion FSO_2^-.

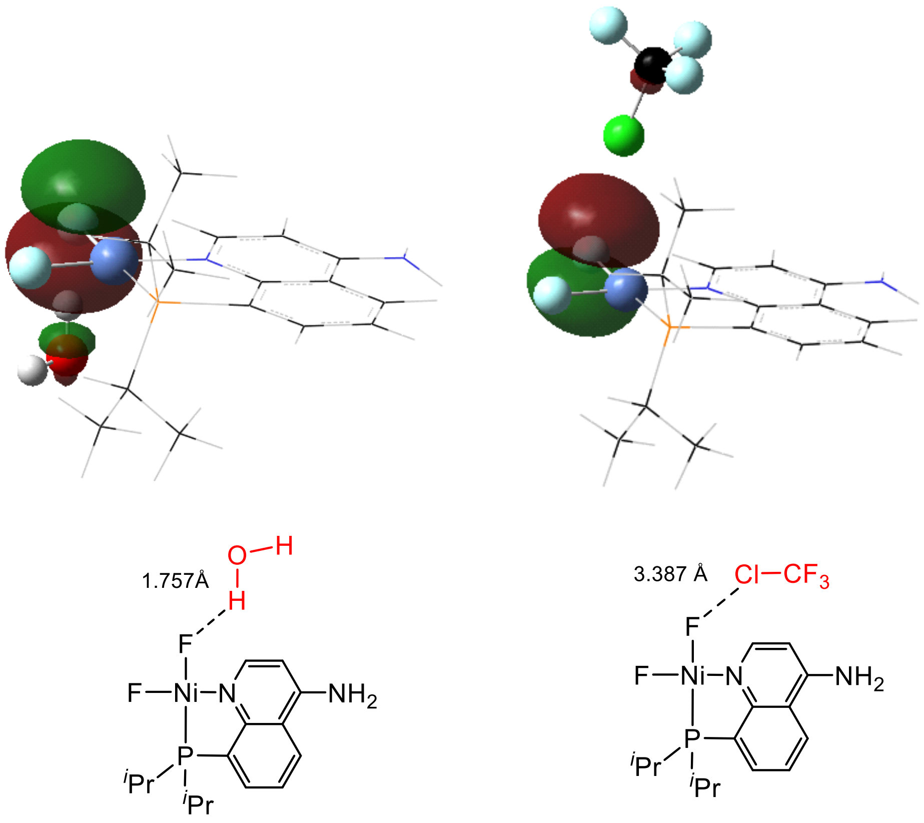

1_H$_2$O_a **1_CF$_3$Cl_a**

Figure S6. The natural localized molecular orbitals (NLMOs) analysis on the F···H and F···Cl interactions in **1_H$_2$O_a** and **1_CF$_3$Cl_a**.

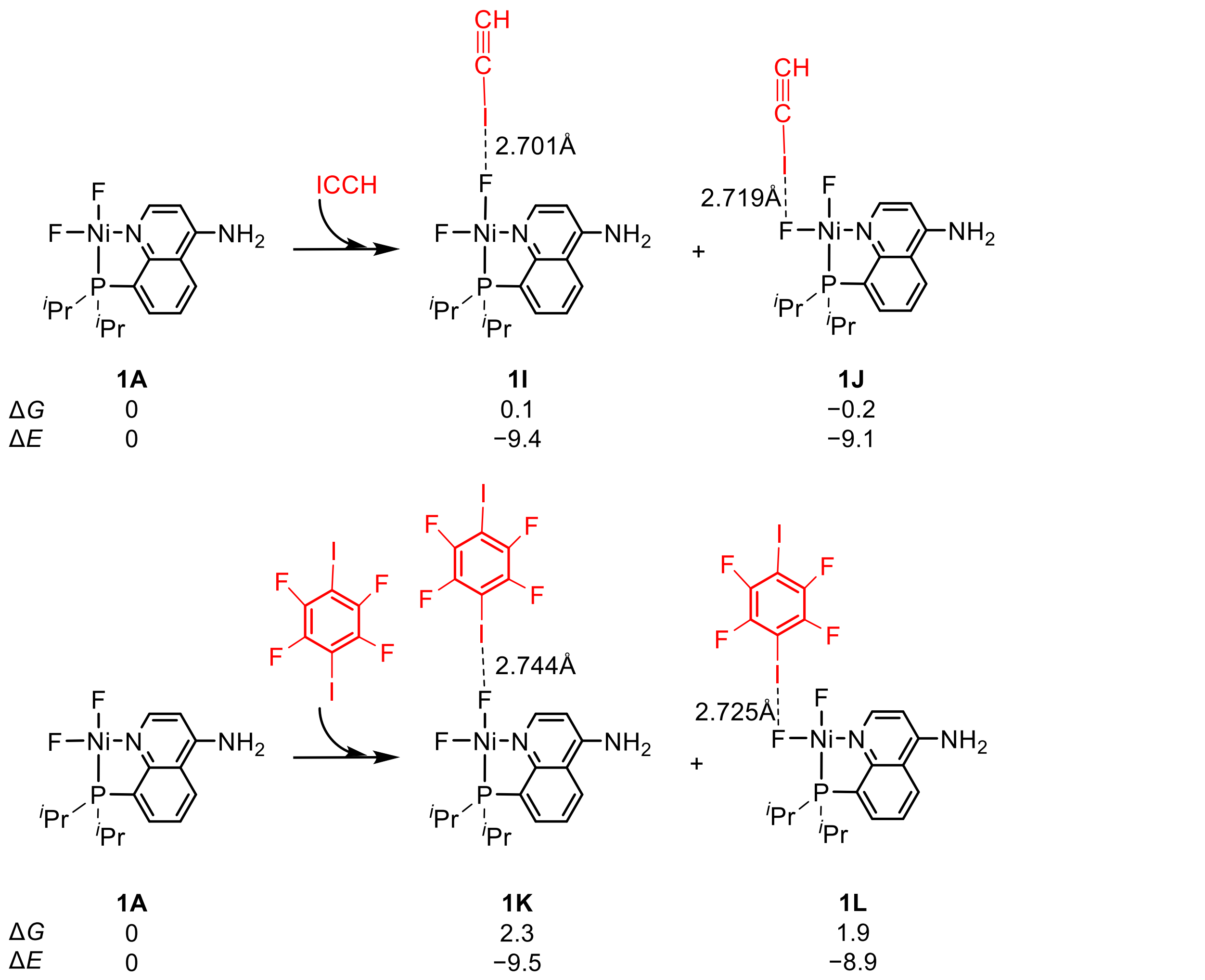

Figure S7. The formation of halogen bond complexes between the structure **1A** and other halogen bond donors. The relative Gibbs free energies (Δ*G*) and potential energies (Δ*E*) are in kcal/mol.

As shown in Figure S7, the Ni−F model complex **1A** bonds the 1-iodoalkyne molecule by halogen bond interaction, resulting in halogen-bonded complexes **1I** and **1J** with free energies of 0.1 and −0.2 kcal/mol, respectively. The I···F halogen bond lengths are 2.701 and 2.719 Å, respectively. The Ni−F model complex **1A** bonds the 1,4-diiodotetrafluorobenzene molecule through halogen bond interactions, resulting in halogen-bonded complexes **1K** and **1L** with free energies of 2.3 and 1.9 kcal/mol, respectively. The I···F halogen bond lengths are 2.744 and 2.725 Å, respectively.

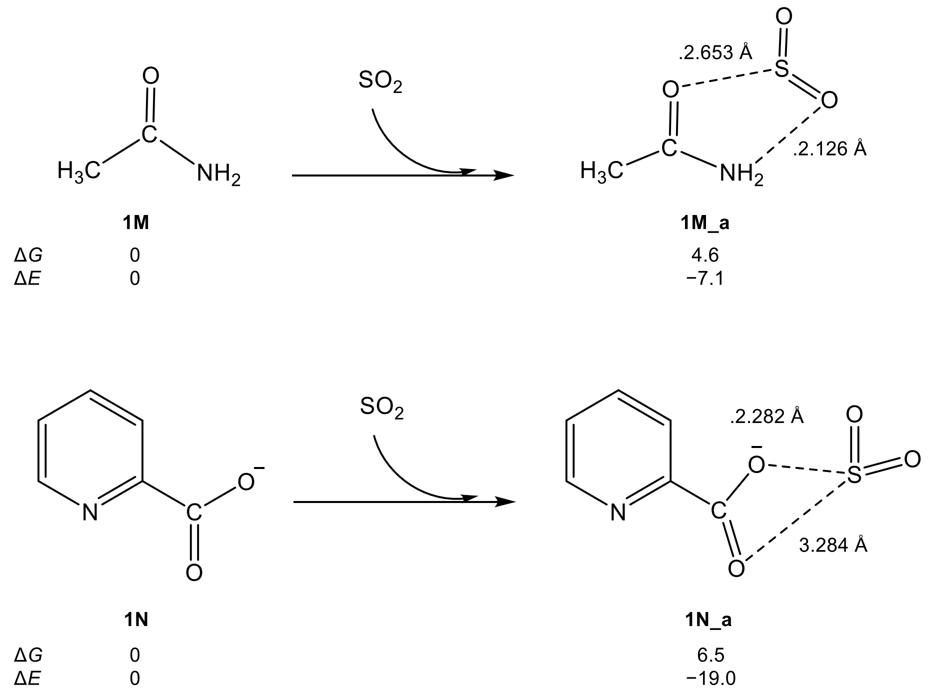

Figure S8. The formation of chalcogen bond complexes between the structure **1M**, **1N** and SO_2. The relative Gibbs free energies (ΔG) and potential energies (ΔE) are in kcal/mol.

As shown in Figure S8, compounds **1M** and **1N** bond the SO2 molecule by sulfur bond interaction, resulting in sulfur bonds **1M_a** and **1N_a** with free energies of 4.6 and 6.5 kcal/mol, respectively. The S···O chalcogen bond lengths are 2.653 and 2.282 Å, respectively.

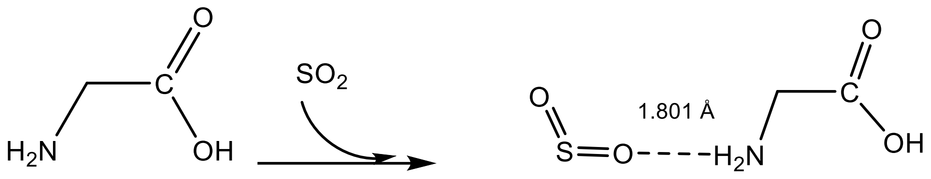

	**1O**	**1O_a**
Δ*G*	0	10.4 kcal/mol
Δ*E*	0	−1.7 kcal/mol

Figure S9. The formation of hydrogen bond complexes between SO_2 and glycine **1O**. The relative Gibbs free energies (Δ*G*) and potential energies (Δ*E*) are in kcal/mol.

As shown in Figure S9, compound **1O** bonds the SO_2 molecule through hydrogen bonding interactions, resulting in hydrogen bonding **1O_a** bonds with a free energy of 10.4 kcal/mol. N−H···O=S hydrogen bond length is 1.801 Å.

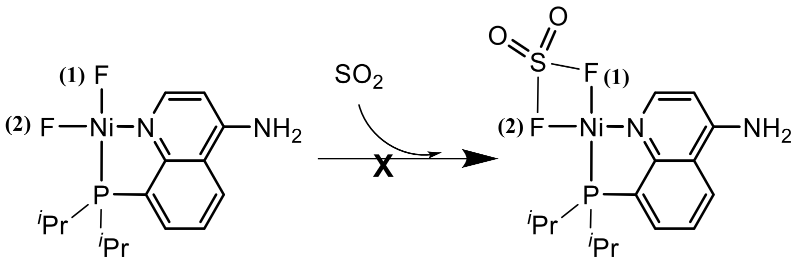

1A **Not available**

Figure S10. Modeling the bidentate coordination of SO_2 with two fluoride atoms. The relative Gibbs free energies (ΔG) and potential energies (ΔE) are in kcal/mol.

We have examined the reaction mode of SO_2 as a bidentate ligand, but this complex can not be located suggesting it is not local minimal.

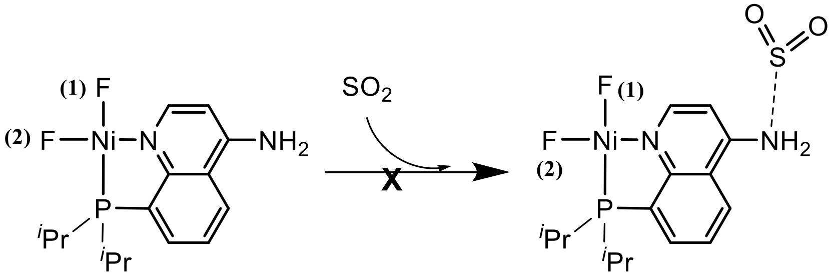

1A

Not available

Figure S11. The nitrogen atom of the $-NH_2$ group as absorption sites. The relative Gibbs free energies (ΔG) and potential energies (ΔE) are in kcal/mol.

We have considered the lone pairs of electrons on the nitrogen atom of the $-NH_2$ group as absorption sites. The $-NH_2$ group cannot serve as the substitution site for SO_2

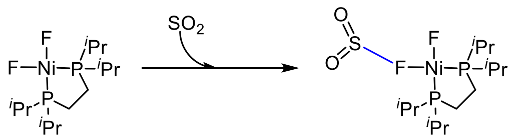

2A_^{i}Pr → **2B_^{i}Pr**

	**2A_^{i}Pr**	**2B_^{i}Pr**
ΔG	0	−1.9
ΔE	0	−10.9

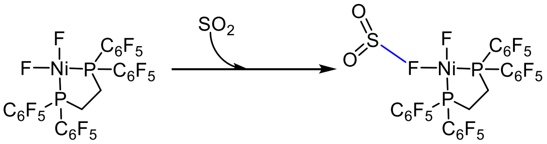

	**2A_C$_6$F$_5$**	**2B_C$_6$F$_5$**
ΔG	0	−1.3
ΔE	0	−11.5

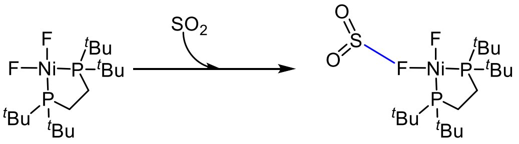

	**2A_^{t}Bu**	**2B_^{t}Bu**
ΔG	0	−0.7
ΔE	0	−12.6

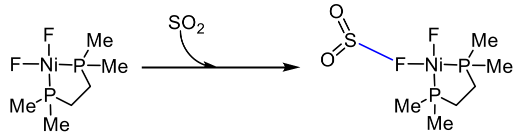

	**2A_Me**	**2B_Me**
ΔG	0	−0.4
ΔE	0	−12.8

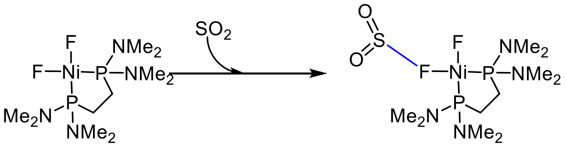

	**2A_NMe**	**2B_NMe**
ΔG	0	−0.2
ΔE	0	−10.4

(a)

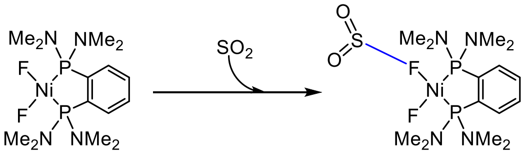

	**3A_NMe**	**3B_NMe**
ΔG	0	−1.0
ΔE	0	−12.5

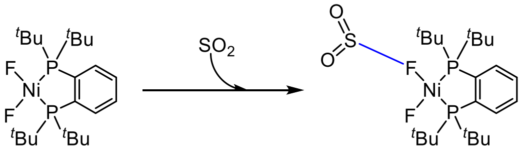

	**3A_^{t}Bu**	**3B_^{t}Bu**
ΔG	0	−0.6
ΔE	0	−12.8

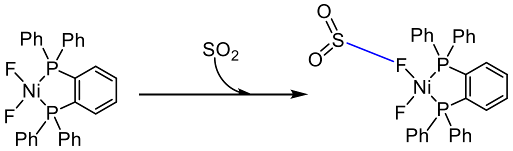

	**3A_Ph**	**3B_Ph**
ΔG	0	−0.5
ΔE	0	−11.8

(b)

Figure S12. The absorption of SO_2 by the bisphosphine Ni complexes **2A** and **3A** with different substituents. The relative Gibbs free energies (ΔG) and potential energies (ΔE) are in kcal/mol.

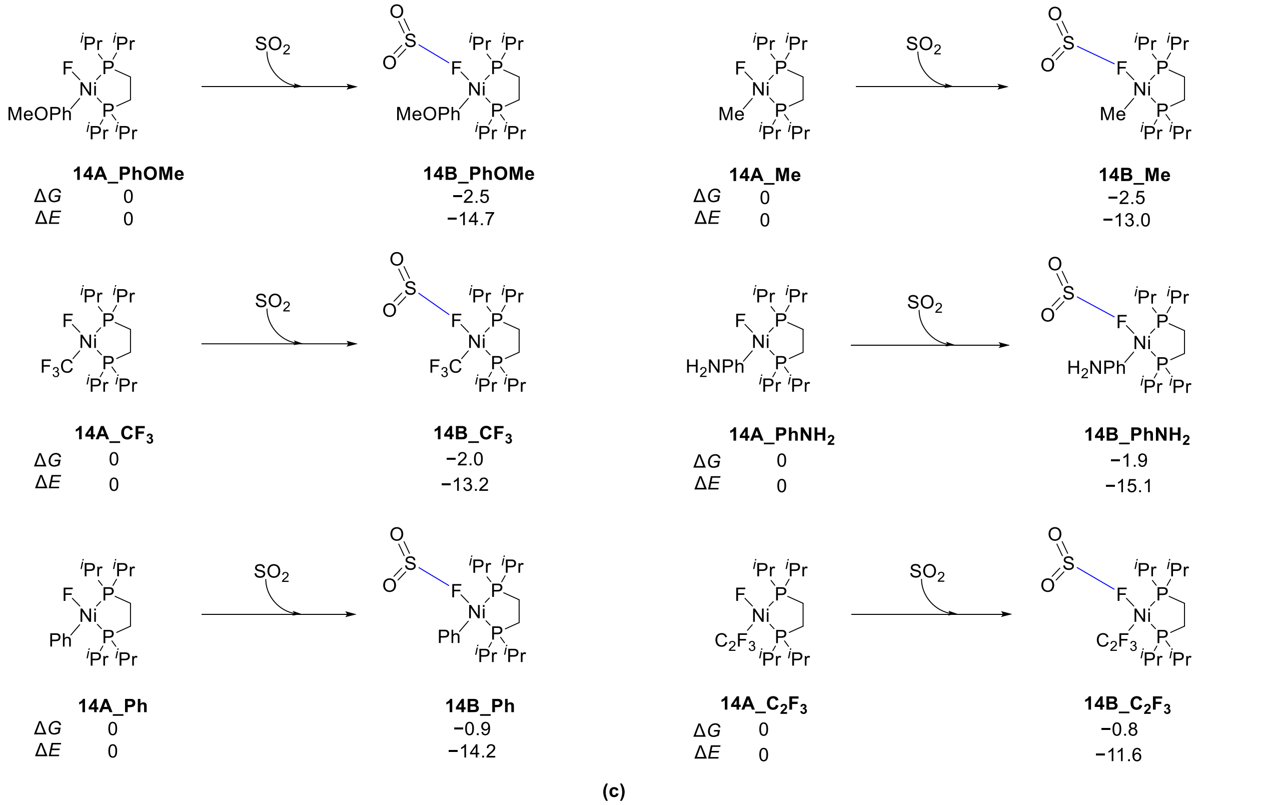

Figure S13. The absorption of SO_2 by the bisphosphine Ni complexes **14A** with different X ligands. The relative Gibbs free energies (ΔG) and potential energies (ΔE) are in kcal/mol.

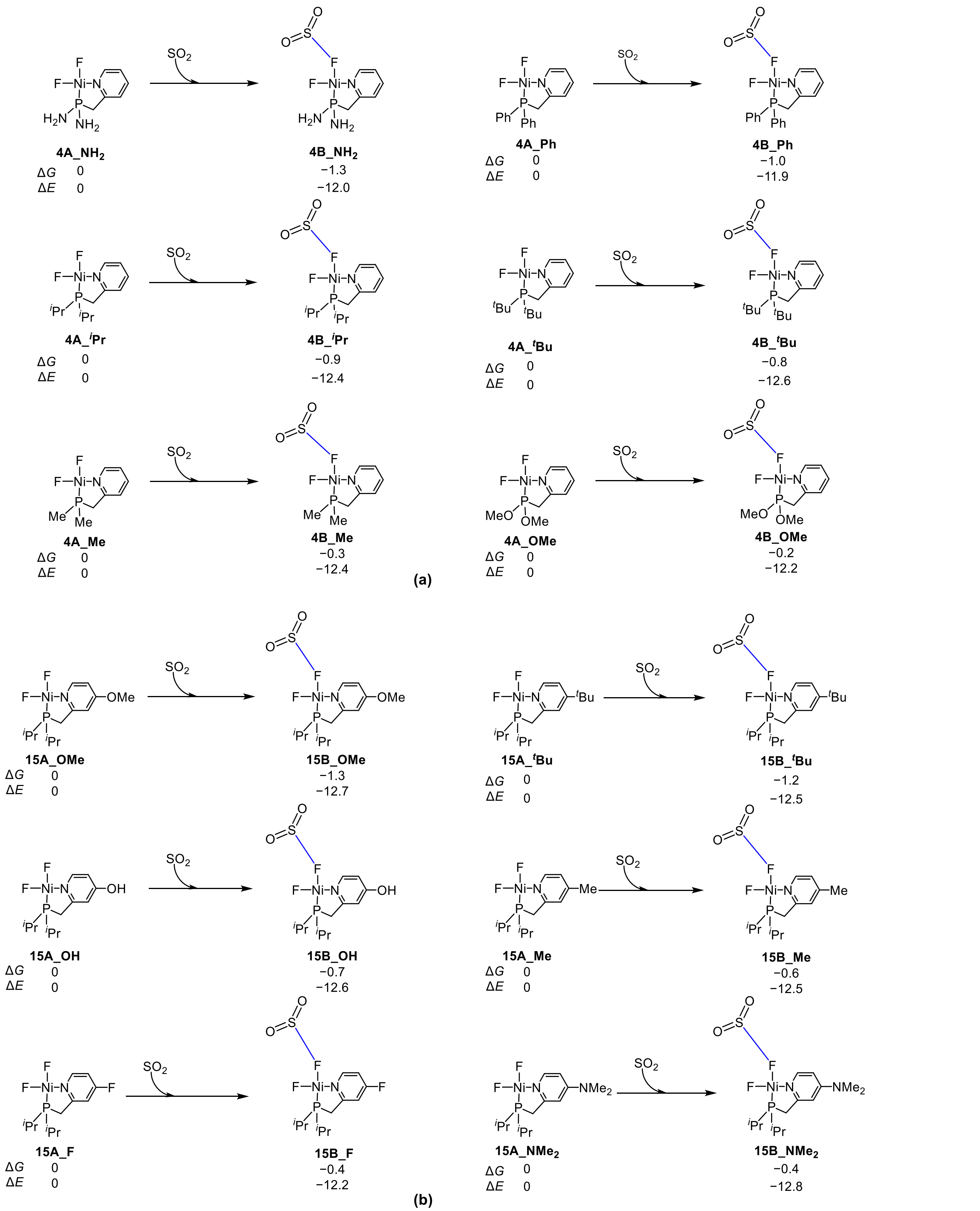

Figure S14. The absorption of SO_2 by the *P,N*-heterobidentate Ni complexes **4A** and **15A** with different substituents. The relative Gibbs free energies (ΔG) and potential energies (ΔE) are in kcal/mol.

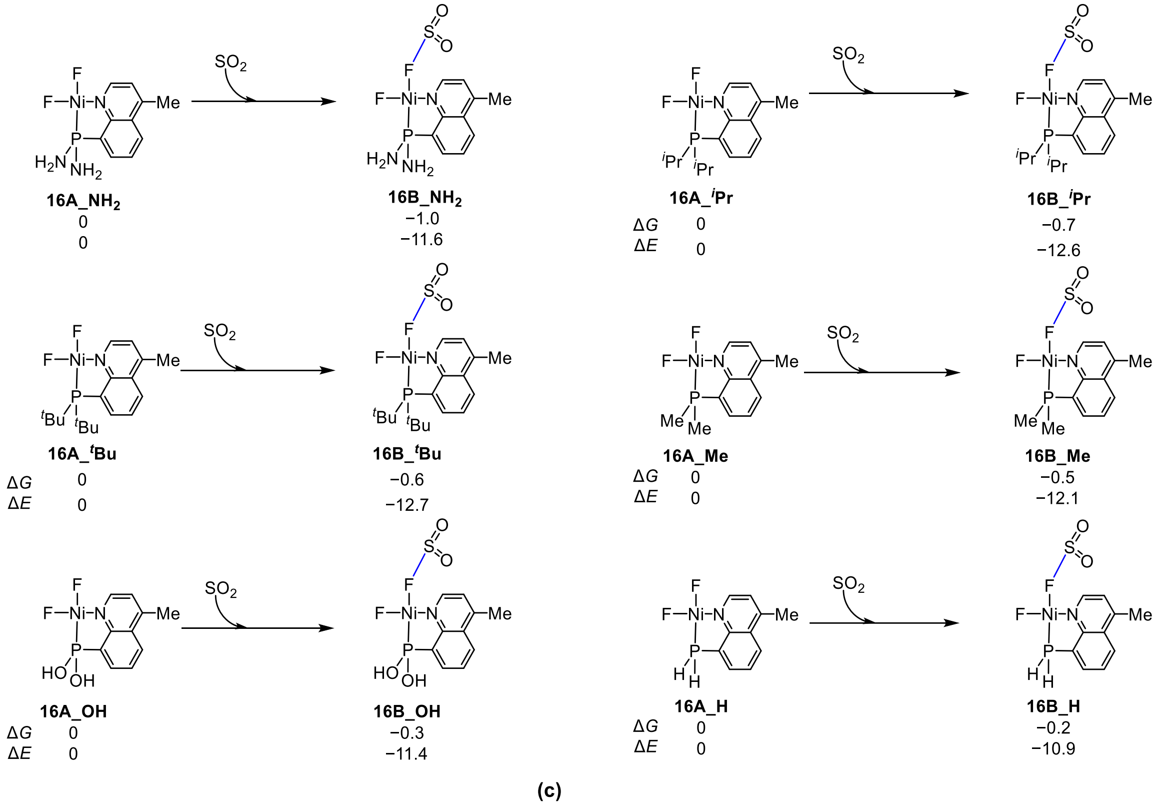

Figure S15. The absorption of SO_2 by the *P,N*-heterobidentate ligand Ni complexes **16A** with different substituents. The relative Gibbs free energies (ΔG) and potential energies (ΔE) are in kcal/mol.

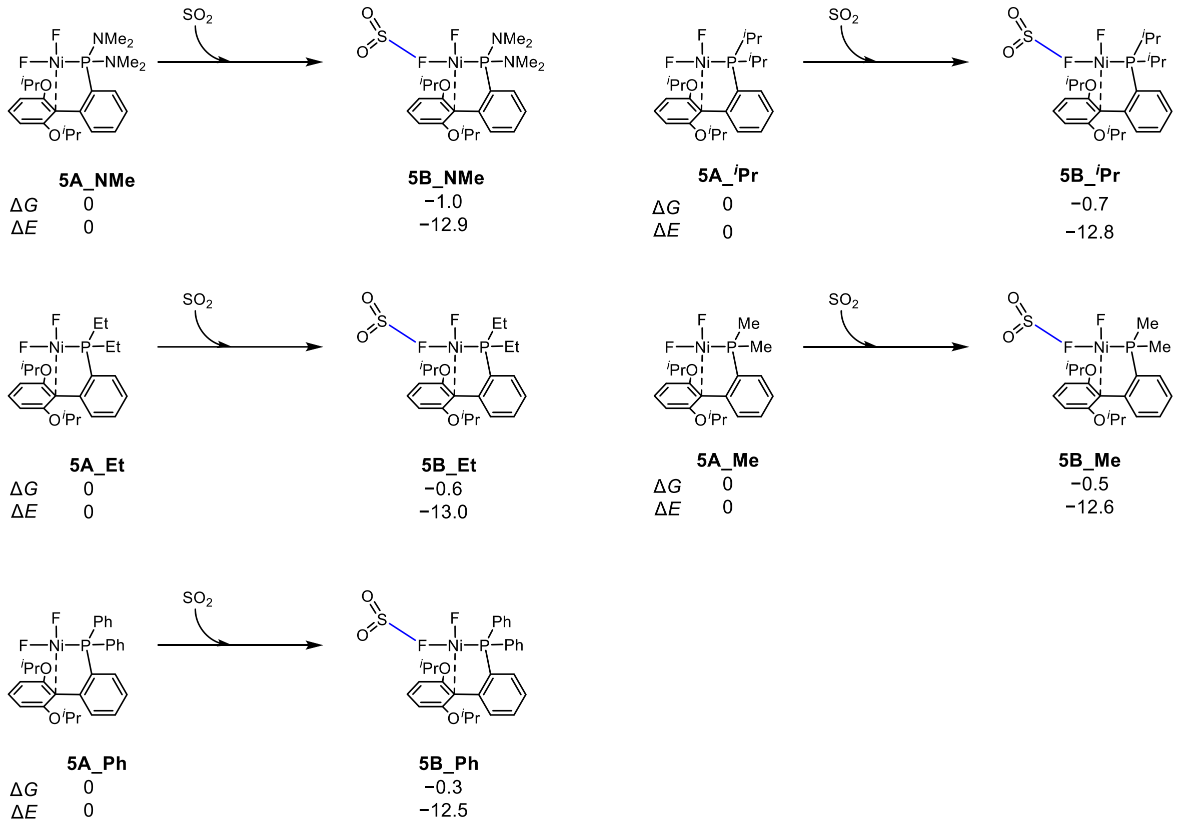

Figure S16. The absorption of SO_2 by the monodentate ligand Ni complexes **5A** with different substituents. The relative Gibbs free energies (Δ*G*) and potential energies (Δ*E*) are in kcal/mol.

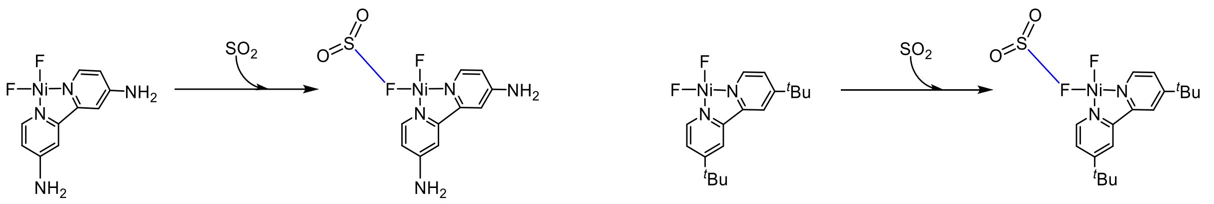

	6A_NH$_2$	6B_NH$_2$	6A_^{t}Bu	6B_^{t}Bu
Δ*G*	0	−0.8	0	−0.5
Δ*E*	0	−11.7	0	−11.3

(a)

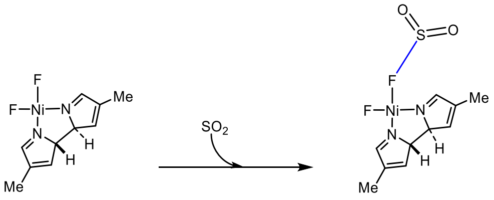

	17A_Me	17B_Me
Δ*G*	0	−0.7
Δ*E*	0	−12.1

(b)

Figure S17. The absorption of SO_2 by the *N,N*-heterobidentate ligand Ni complexes **6A** and **17A** with different substituents. The relative Gibbs free energies (Δ*G*) and potential energies (Δ*E*) are in kcal/mol.

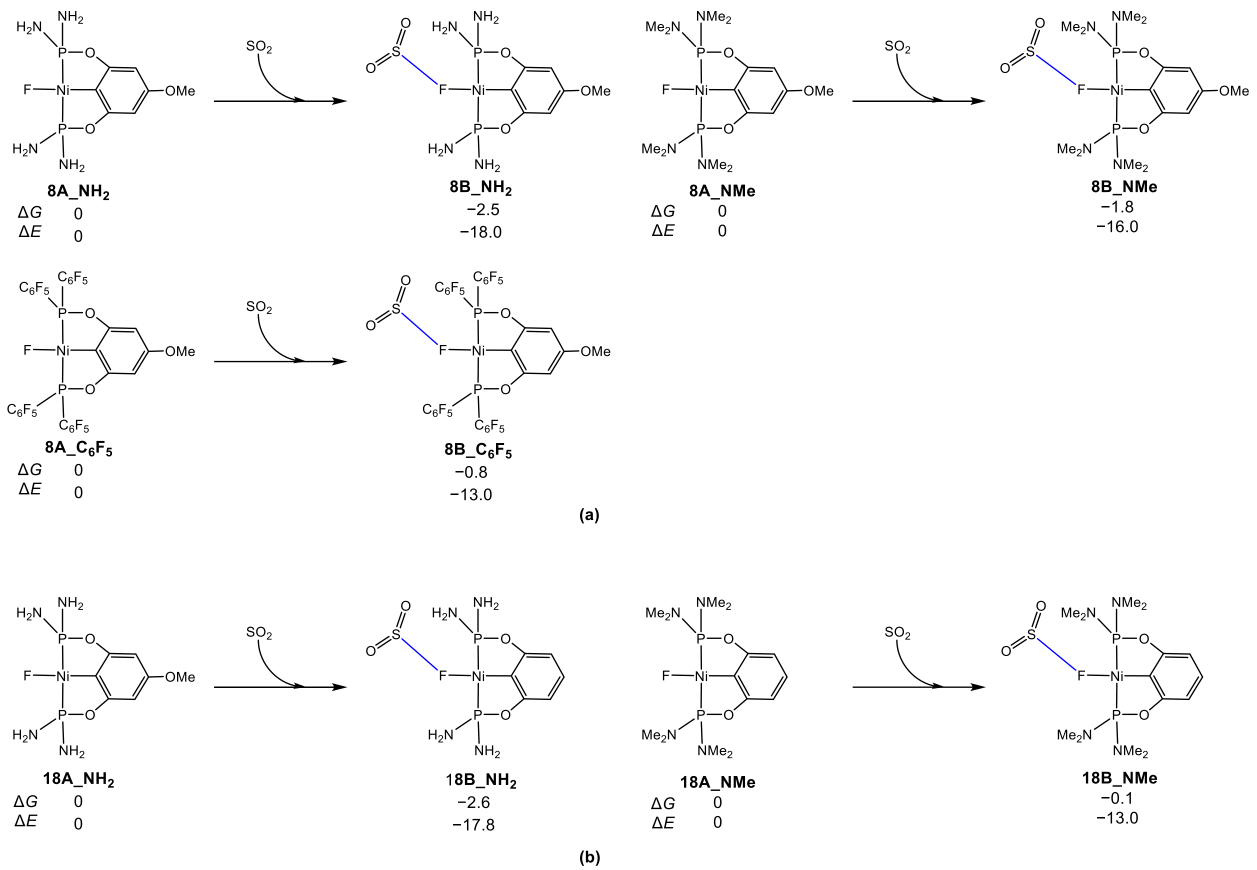

Figure S18. The absorption of SO_2 by the pincer ligand Ni complexes **8A** and **18A** with different substituents. The relative Gibbs free energies (ΔG) and potential energies (ΔE) are in kcal/mol.

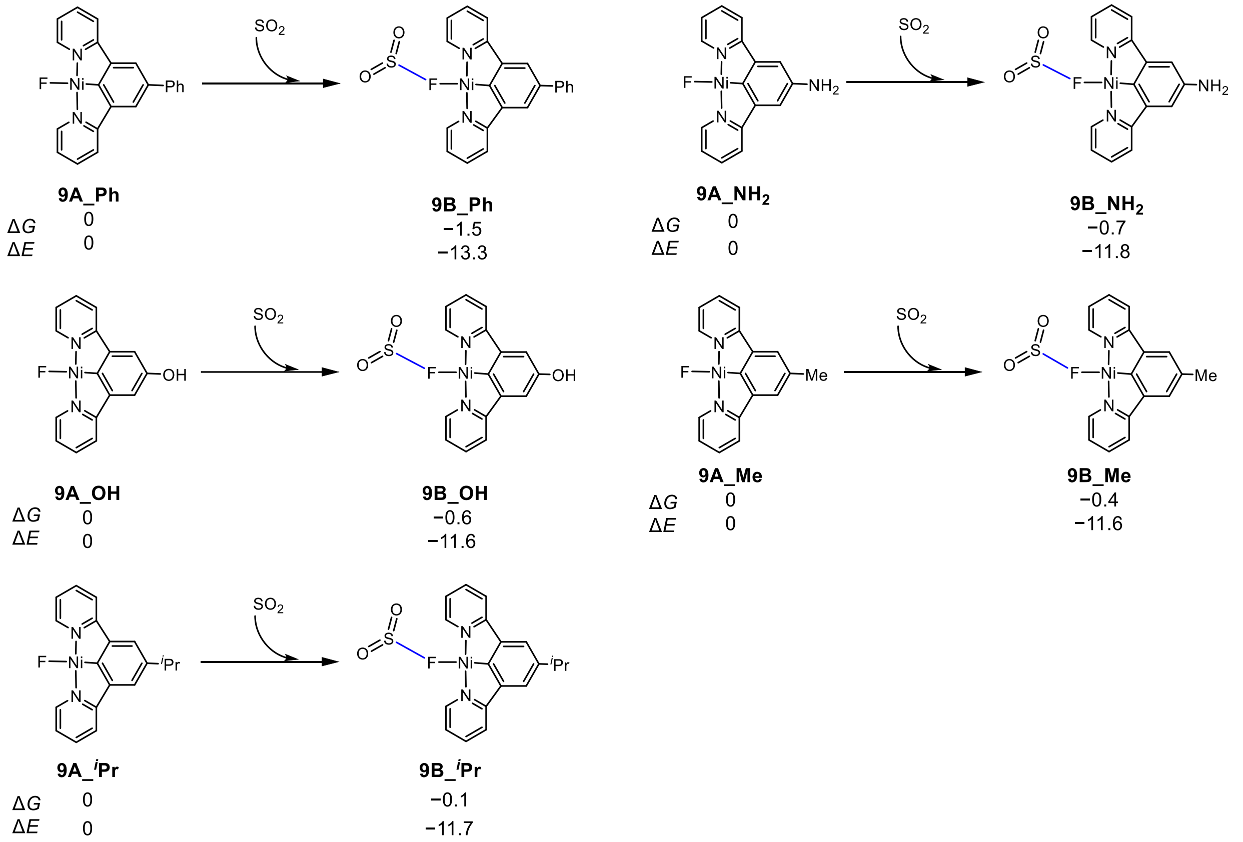

Figure S19. The absorption of SO_2 by the pincer ligand Ni complexes **9A** with different substituents. The relative Gibbs free energies (ΔG) and potential energies (ΔE) are in kcal/mol.

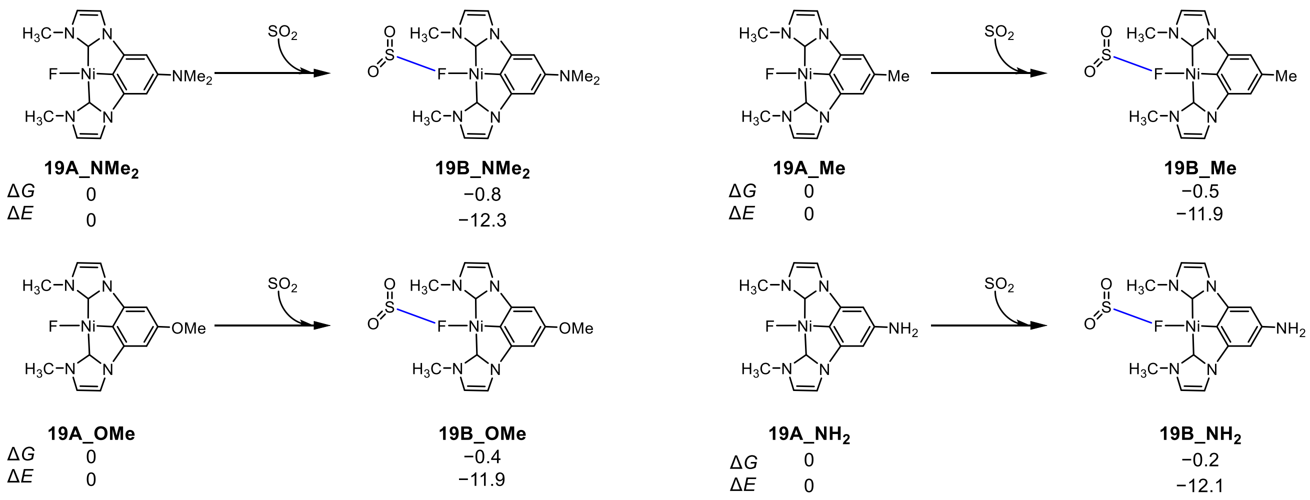

Figure S20. The absorption of SO_2 by the pincer ligand Ni complexes **19A** with different substituents. The relative Gibbs free energies (ΔG) and potential energies (ΔE) are in kcal/mol.

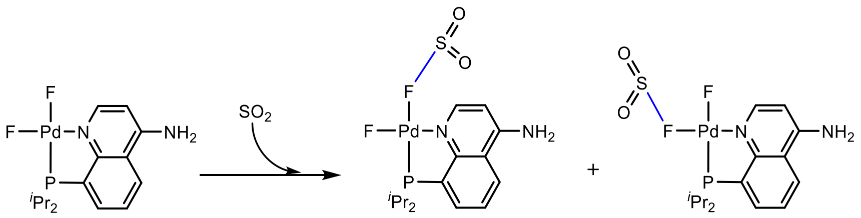

	1A_Pd	1B_Pd	1C_Pd
Δ*G*	0	−2.4	−0.1
Δ*E*	0	−13.8	−12.7

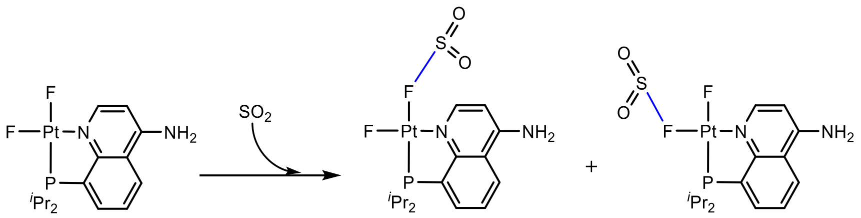

	1A_Pt	1B_Pt	1C_Pt
Δ*G*	0	−0.8	1.5
Δ*E*	0	−12.7	−12.2

Figure S21. The comparisons of the Pd and Pt metals for the absorption of SO_2 by complex **1A**. The relative Gibbs free energies (Δ*G*) and potential energies (Δ*E*) are in kcal/mol.

$$\Delta G = -RT \ln K$$

$$-2.2 * 4184 \text{ J} / mol = -8.314 \text{ J} / \left(mol \bullet K \right) * 298 K * \ln K$$

$$K = \frac{\alpha}{1 - \alpha}$$

$$\alpha = 97.6\%$$

Figure S22. The calculation of the conversion rate for the formation of the complex **1B**.
